# Telomere maintenance through recruitment of internal genomic regions

**DOI:** 10.1038/ncomms9189

**Published:** 2015-09-18

**Authors:** Beomseok Seo, Chuna Kim, Mark Hills, Sanghyun Sung, Hyesook Kim, Eunkyeong Kim, Daisy S. Lim, Hyun-Seok Oh, Rachael Mi Jung Choi, Jongsik Chun, Jaegal Shim, Junho Lee

**Affiliations:** 1 Department of Biological Sciences, Institute of Molecular Biology and Genetics, Seoul National University, Seoul 08826, Korea; 2 Terry Fox Laboratory, BC Cancer Agency, Vancouver, Canada V5Z 1L3; 3 Department of Biological Sciences, Bioinformatics Institute, BIO-MAX, Seoul National University, Seoul 08826, Korea; 4 Research Institute, National Cancer Center, Goyang, Gyeonggi 10408, Korea; 5 Department of Biophysics and Chemical Biology, Seoul National University, Seoul 08826, Korea

## Abstract

Cells surviving crisis are often tumorigenic and their telomeres are commonly maintained through the reactivation of telomerase. However, surviving cells occasionally activate a recombination-based mechanism called alternative lengthening of telomeres (ALT). Here we establish stably maintained survivors in telomerase-deleted *Caenorhabditis elegans* that escape from sterility by activating ALT. ALT survivors *trans*-duplicate an internal genomic region, which is already *cis*-duplicated to chromosome ends, across the telomeres of all chromosomes. These ‘Template for ALT' (TALT) regions consist of a block of genomic DNA flanked by telomere-like sequences, and are different between two genetic background. We establish a model that an ancestral duplication of a donor TALT region to a proximal telomere region forms a genomic reservoir ready to be incorporated into telomeres on ALT activation.

## Abstract (web summary)

Telomeres in post-crisis cells are maintained by re-activated telomerase or by alternative lengthening of telomeres (ALT). Here, Seo *et al*. report a stable mode of ALT in worm where internal genomic regions generate a genomic reservoir ready to be incorporated into telomeres upon ALT activation.

## Graphical abstract

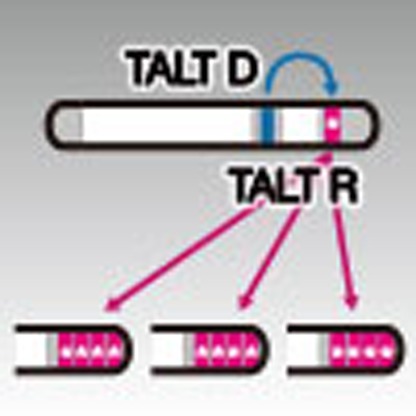

The end-replication problem imposes a replicative limit for linear chromosomes^1^, and replication beyond this limit leads to crisis and catastrophic cell death. Rare cell survivors are able to overcome crisis by regaining a telomere maintenance mechanism but often show chromosomal instability and are ultimately tumorigenic. These cells maintain telomere length most commonly through the reactivation of telomerase^2^ but occasionally activate the recombination-based mechanism alternative lengthening of telomeres (ALT)^3^. Although specific genes are reportedly important for the maintenance of ALT^4^,^5^,^6^, it is largely unknown how specific tumours activate ALT rather than telomerase in maintaining telomere length.

To investigate the mechanism underlying ALT activation, we used a genetic model of the nematode *Caenorhabditis elegans* in which the telomerase gene *trt-1* is deleted^7^ such that telomere-mediated sterility occurs after 14–18 generations. In this model system, survivors that are maintained beyond the expected number of generations can arise only through a telomerase-independent ALT mechanism, enabling us to investigate the ALT mechanism by genetically preventing telomerase activation.

We show that the stably maintained survivor lines utilize an internal genomic region as a template for telomere lengthening. We define two such ‘Template for ALT' (TALT) regions, both of which share a common sequence structure consisting of a block of genomic DNA flanked by telomere-like sequences. We also show these regions have *cis*-duplicated to telomeric ends, forming reservoirs that are incorporated into telomeres on ALT activation. The TALTs we report here represent a novel feature of ALT, whereby internal genomic regions are co-opted to protect chromosome ends in the absence of telomerase. We also discuss the evolutionary implications of our findings.

## Results

### Isolation of stable ALT survivors of *C. elegans*

**Figure 1:**
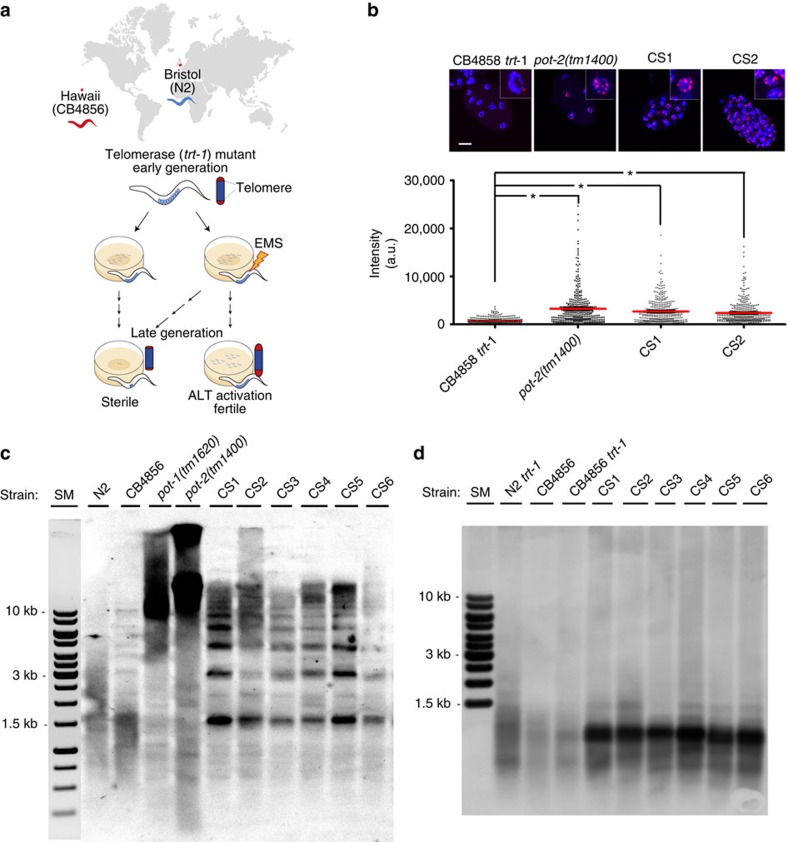
Isolation of stable ALT survivors in *C. elegans* with distinct telomeric sequences. (**a**) A schematic diagram showing the experimental procedures to isolate stable ALT survivors in two wild isolates. (**b**) Quantitative FISH analysis for telomere length of the survivors CB4856 *trt-1*, *pot-2(tm1400)*, CS1 and CS2. Telomere was detected by Cy3-TTAGGC*3 PNA probe (red) in the embryo. DNA was counterstained with 4′,6-diamidino-2-phenylindole (blue). The upper panel shows representative images for each strain. *t*-test was used for statistical analysis for quantification (*P value <0.0001, *n*=388/each). Mean value is represented with red bar. Scale bar, 10μm. (**c**) TRF analysis of N2, CB4856, *pot-1(tm1620)*, *pot-2(tm1400)* and all the CS survivors digested with a combination of six-cutter restriction endonucleases (*Nhe*I, *Dra*I, *Apa*I, *Nde*I, *Xho*I, *Nco*I and *Sac*I) and probed with DIG-TTAGGC*4. *pot-1(tm1620)* and *pot-2(tm1400)* were used as positive controls as they have long telomeres. (**d**) TRF analysis of CB4856, CB4856 *trt-1* and all the CS survivors probed with DIG-TTAGGC*4 after *Hinf*I digestion.

A deletion allele of the telomerase gene *trt-1*, *trt-1(ok410)*, was introduced into wild-type N2 and CB4856 worm backgrounds, strains that show an extensive divergence in single-nucleotide variants (SNVs)^8^. We treated these worms with a DNA-alkylating agent ethyl methane sulfonate (EMS), and after eight generations, isolated survivors (Fig. 1a and Supplementary Fig. 1a,c,d). We isolated six independent survivors (named as CS1–CS6) from 80 EMS-treated plates of CB4856 *trt-1* worms, while five survivors (named as NS1–NS5) were isolated from 200 EMS-treated plates of N2 *trt-1* worms. All six CS lines and two of the NS lines (NS1 and NS2) were maintained with no gross phenotype for at least 300 generations by transferring 10–15 individuals every generation, suggesting that these ALT lines are stably maintained. The remaining three NS survivors (NS3–NS5) were maintained only by massive transfer of worms every generation (Supplementary Fig. 1b). The chromosome number of all the ALT survivors decreased due to chromosomal fusions, suggesting that telomeres were critically shortened before ALT was established (Supplementary Fig. 1e).

Similar to ALT in humans, the telomere lengths of CS survivors were longer than those of in the starting strains when assessed by both fluorescent *in situ* hybridization (FISH) and terminal restriction fragment (TRF) analysis as well as whole-genome sequencing (WGS) analysis (Fig. 1b,c and Supplementary Figs 2a and 5a). The telomere signal was abolished after BAL 31 exonuclease treatment (Supplementary Fig. 2b), suggesting that they were located at chromosomal ends. Surprisingly, unlike the starting CB4856 *trt-1* strain, all CS survivor lines showed discrete banding patterns on a TRF analysis when the *Hinf*l restriction enzyme was used (Fig. 1d and Supplementary Fig. 5b). Since the recognition sequence for this enzyme is not present in the canonical *C. elegans* telomere repeat, these data suggested that additional sequences were interspersed within the telomere. We therefore hypothesized that CS survivors were able to maintain genomic integrity by lengthening their telomeres with sequences different from the canonical telomeres.

### Telomere maintenance without mutations in CS survivors

Since we used EMS, a known potent mutagen, to induce ALT survivors, we wanted to identify all specific mutations to find those responsible for the ALT phenotype in the CB4856 *trt-1* background. To do this, we analysed the whole-genome sequences of the CS1 and CS2 survivor lines, before and after multiple rounds of outcross with N2 *trt-1* worms to exclude genetic variations unrelated to the ALT phenotype. None of the variants that had been present in the initial isolates were maintained after outcrosses in the CS1 and CS2 survivor lines, suggesting that no point mutation was responsible for ALT activation (Supplementary Fig. 3a,b). In addition, we were unable to induce ALT by RNAi of the genes identified as small indels by array comparative genome hybridization (Supplementary Fig. 3c,d). Combined, while we cannot rule out the possibility that there was an ALT-initiating mutation that was subsequently not required for maintenance of ALT, it is more likely that survivors acquired potentially more complex genomic events, which were responsible for ALT induction after EMS treatment.

### Telomere maintenance by TALT1 in CS survivors

**Figure 2:**
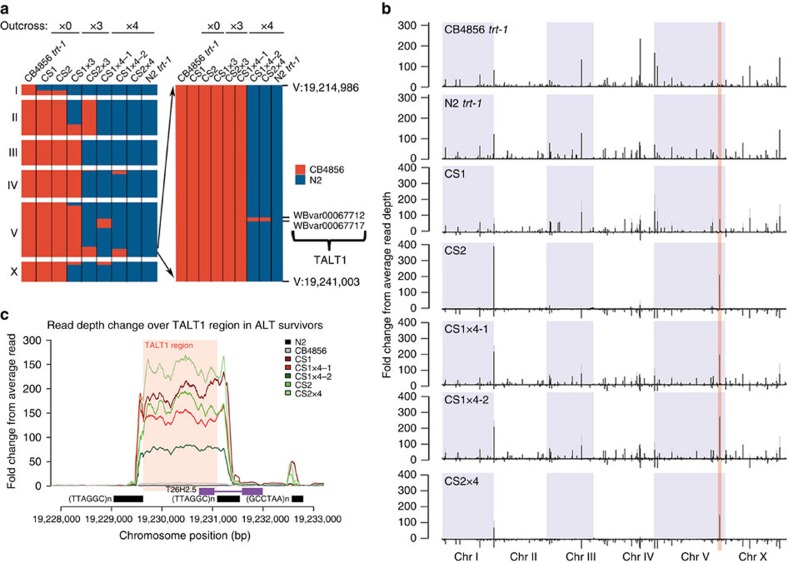
Identification of template for ALT in CB4856 backgrounds. (**a**) Association heat map of CB4856 SNVs that were retained after extensive rounds of outcrosses. The right panel is an enlargement image near the TALT1 locus on chromosome (Chr) V. (**b**) Fold change from average read depth plot (black bar) of CB4856 *trt-1*, N2 *trt-1*, CS1, CS2 and outcrossed CS survivors with N2 (CS1x4-1, CS1x4-2 and CS2x4). To normalize strain-specific variation, fold change from average depth of parental CB4856 *trt-1* was subtracted from total fold change (grey bar) of ALT survivors. While positive value indicates over-representation of the sequence, negative value indicates under-representation of the sequence compared with control. (**c**) An enlargement of TALT1 peak on chromosome V from **b** (red shade). Internal telomere repeats are indicated by black bars. Gene structure flanking TALT1 locus is denoted by purple bar.

To identify specific genomic regions that are closely associated with ALT induction from the starting strain of CB4856 *trt-1*, we employed an association mapping strategy. We reasoned that if any specific genomic region conferred the ALT phenotype, the CB4856 *trt-1* survivors should retain this region, even after extensive outcrosses with genetically distinct N2 *trt-1* worms. We therefore identified all CB4856-specific regions that were not erased by successive outcrosses with N2 *trt-1* in the genomes of both CS1 and CS2 survivors. We found a single region on chromosome V that contained the only two CB4856 *trt-1*-derived SNVs retained after multiple outcrosses, suggesting this region is tightly associated with the ALT phenotype both in CS1 and CS2 survivors (Fig. 2a).

Further analysis of the WGS data of CS1 and CS2 survivors showed that a specific genomic region of 1,464 bp (Chr V: 19,229,626–19,231,090) was over-represented in the CS1 and CS2 survivors as high as 200-fold above the average sequence depth (Fig. 2b). This element, which we termed ‘TALT1', remained the most amplified region even after extensive outcrosses (Fig. 2b and Supplementary Fig. 4a). In addition, the relative copy number of TALT1 increased after outcrosses compared with that of initial survivors, suggesting that TALT1-containing telomeres are actively replicated in the survivors. The genomic structure of TALT1 consisted of a block of uniquely mapping sequence containing the promoter and the first exon of a coding gene, T26H2.5, flanked by two interstitial telomere-like sequences (ITS), present in the same orientation (Fig. 2c). TALT1 was found to be amplified in all six CS survivors (Supplementary Fig. 4b). To determine the locations of the multiple copies of TALT1 in the genome of the survivors, we extracted all paired-end sequence reads that mapped to TALT1, and used overlapping mate pairs to construct contiguous fragments ranging from 200 bp to 1.7 kb long.

**Figure 3:**
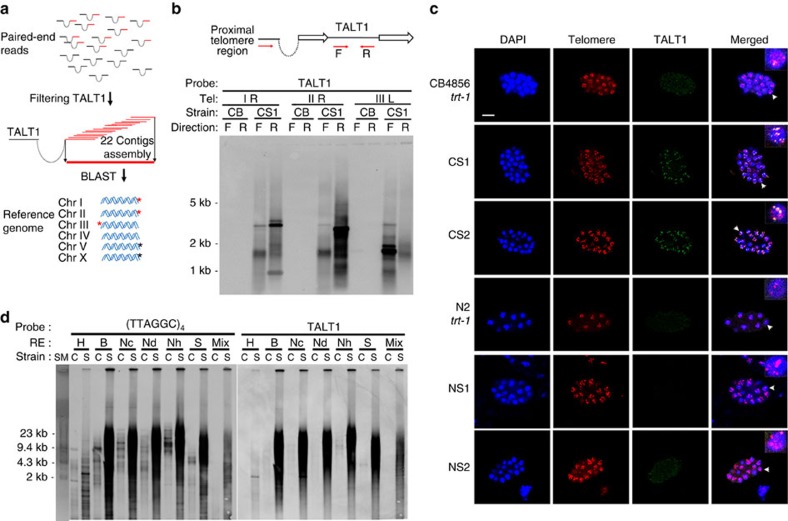
TALT1 is replicated in the telomeres of CB4856 survivors. (**a**) Diagram illustrating how the contigs (red bar) were reconstructed from reads in which one pair aligned to TALT1. Asterisks indicate predicted breakpoint by BLAST (black) or confirmed breakpoints (red). (**b**) Confirmation of *trans*-duplication by breakpoint PCR followed by Southern hybridization with TALT1 probe. Breakpoint PCR primers (red arrows) were designed to amplify the junction of TALT1 and each of proximal telomere regions. F, forward primer; R, reverse primer; open arrow, telomere repeat. (**c**) FISH using the TALT1 probe and telomere probe in embryonic stage. Scale bar, 10μm; blue, 4′,6-diamidino-2-phenylindole; green, TALT1; red, telomere. (**d**) TRF analysis using a TALT1 probe and a telomere probe. DNA was digested with indicated enzymes and hybridized with indicated probes. RE, restriction endonuclease; C, CB4856 *trt-1*; S, CS1 survivor; H, *Hind*III; B, *Bam*HI; Nc, *Nco*I; Nd, *Nde*I; Nh, *Nhe*I; S, *Sac*I; Mix, mixure of *Hind*III, *Bam*HI, *Nco*I, *Nde*I, *Nhe*I and *Sac*I.

We generated five contigs that spanned both TALT1 and specific chromosome ends (Fig. 3a and Supplementary Table 1). PCR reactions using primers specific to TALT1 with primers specific to these chromosomal ends showed that amplicons were produced only from the survivor lines (Fig. 3b and Supplementary Table 2). We further confirmed these data with FISH experiments, where only in CS survivors a TALT1 probe co-localized perfectly with a telomere probe (Fig. 3c). Finally, we performed TRF analyses using enzymes lacking restriction sites in TALT1 and compared them with enzymes that cut within the TALT1 sequence. Using both a telomere probe and a TALT1 probe, we assessed the telomere lengths from the TRFs and found they were longer when TALT1 was uncut (Fig. 3d and Supplementary Fig. 5c), conclusively showing that TALT1 is present in multiple copies in the telomeres of the survivors. We further validated that TALT1 was tandemly integrated into the telomeres of CS survivors by PCR amplifying between TALT1 elements (Supplementary Fig. 4c).

### Telomere maintenance by TALT2 in NS survivors

**Figure 4:**
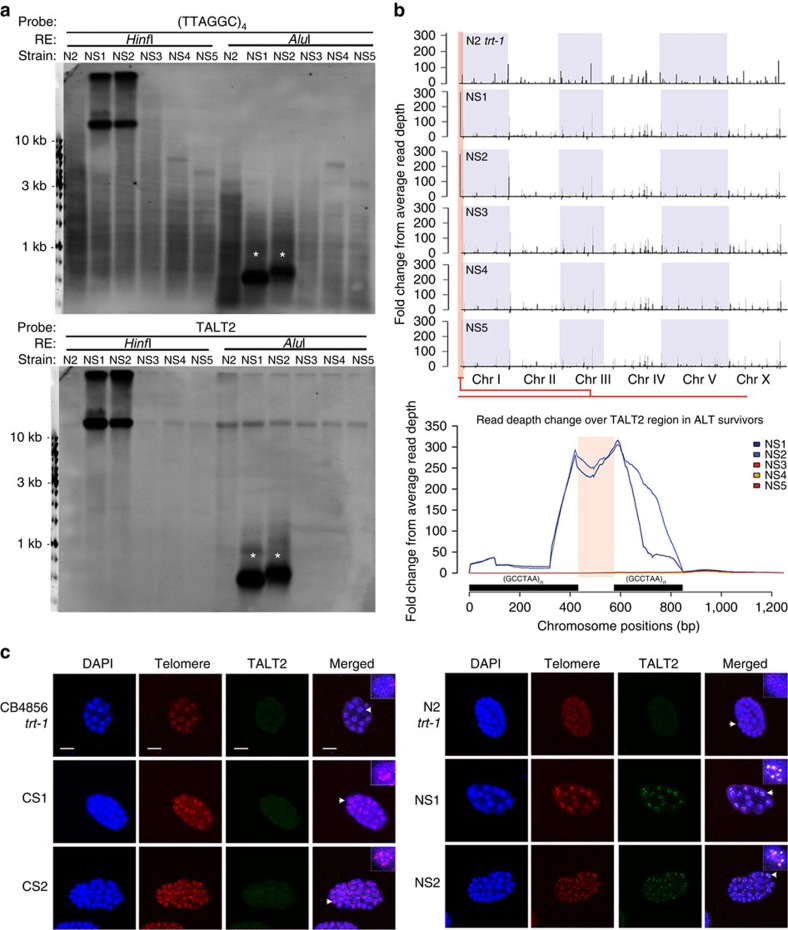
NS survivors amplify and incorporate TALT2 in their telomeres. (**a**) TRF analysis for telomere and TALT2 of N2 survivors. Genomic DNA of N2 *trt-1* and NS survivors (NS1–NS5) was digested with HinfI or AluI. The blot was hybridized with (TTAGGC)*4 repeat (upper panel). The same blot was stripped and reprobed with a TALT2-specific probe (bottom panel). Arrowheads indicate cleaved TALT2. (**b**) Fold change from average read depth plot (black bar) of N2 *trt-1* and N2 survivors. To normalize strain-specific variation, fold change from average depth from parental N2 *trt-1* was subtracted from total fold change (grey bar) of ALT survivors. The bottom panel is an enlargement of TALT on chromosome (Chr) I. Internal telomere repeats are indicated as black bars. (**c**) FISH using the TALT and telomere probe in embryo of CB4856 *trt-1*, N2 *trt-1* and all the survivors. Scale bar, 10μm; blue, 4′,6-diamidino-2-phenylindole; green, TALT; red, telomere.

The discovery of TALT1 in the CS survivors prompted us to explore whether additional TALT regions existed in the N2 *trt-1*-derived ALT survivors that we isolated. Similar to the CS survivors, we found that NS1 and NS2, but not NS3 to NS5, contained longer telomeres than the starting strain, and produced discrete bands on TRF blots using restriction enzymes that do not cut canonical telomere sequences (Fig. 4a and Supplementary Fig. 5d). These bands were markedly different from identically digested CB4856-derived survivor TRFs, suggesting that while the NS survivors had incorporated specific sequences into their telomeres, these sequences were not TALT1 (Supplementary Fig. 6a,b). WGS analysis of NS1 and NS2 revealed that both lines had amplification of a specific genomic region adjacent to the left telomere of chromosome I (Fig. 4b). We named this region ‘TALT2'. A probe specific to TALT2 co-localized to telomeres only in NS1 and NS2 survivors, assessed both by TRF and FISH analyses (Fig. 4a,c), strongly suggesting that TALT2 was enriched at chromosomal ends in the NS1 and NS2 survivors. PCR experiments using primers specific to TALT2 and chromosomal ends validated these findings (Supplementary Table 2). PCR using just TALT2 primers amplified between TALT2 regions and demonstrated that they were arranged tandemly in the telomeres of NS survivors (Supplementary Fig. 4d). The genomic structure of TALT2 is similar to that of TALT1, in that it consists of a unique DNA fragment flanked by telomeric sequences (an ITS at one end and telomere sequences at the other) (Supplementary Fig. 6c).

### Altered double-strand break responses in ALT survivors

The *C. elegans* strain deficient in the telomerase gene was reported to overcome sterility without telomere lengthening only when maintained as large populations, presumably due to stochastic telomere shortening events occurring within the population^9^. In stark contrast to these data, most survivors in this study incorporated TALT sequences into their telomeres and were stably maintained by means of a typical transfer in which a few worms were transferred to a new plate for culture each generation (Supplementary Fig. 1b). Only the NS3–NS5 lines did not show TALT amplification (Supplementary Fig. 4e), and were maintained only by massive transfer. These lines became sterile within 10 generations (Supplementary Fig. 1b) and had short telomeres (Fig. 4a). One reason for the apparent discrepancy is that we treated the worms with EMS, which might have elevated the DNA damage response in some way. Consistent with this, by comparing the RNA-seq analysis of the survivors across specific gene expression data sets, we found that genes significantly altered in ALT survivors correlated with the expression profile of genes induced by gamma radiation (Supplementary Fig. 7)^10^. This suggests that *C. elegans* ALT survivors maintain altered double-strand break responses, similar to human ALT cells^11^.

### ALT mechanism involves TALT duplication in *cis* and in *trans*

**Figure 5:**
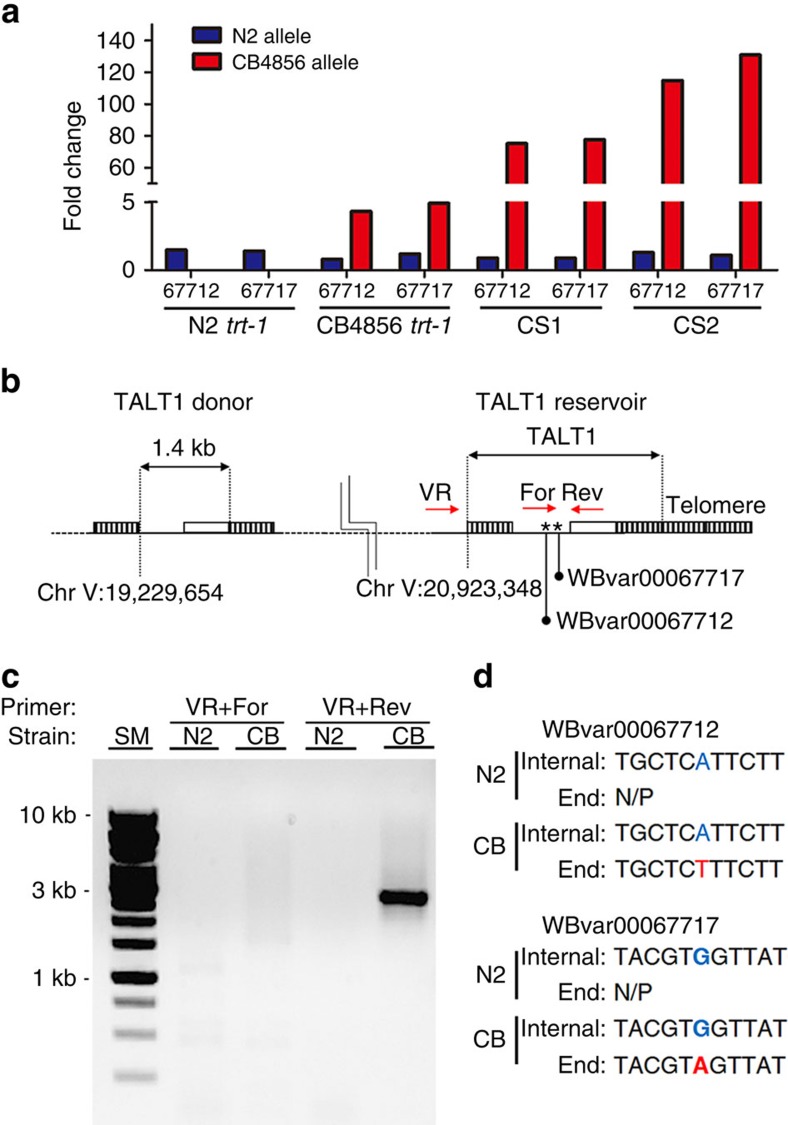
*trans*-Duplication of TALT1 reservoir to other chromosomal ends in CB4856 backgrounds. (**a**) Fold change from average coverage of SNVs (WBvar00067712 and WBvar00067717) from WGS data of N2 *trt-1*, CB4856 *trt-1*, CS1 and CS2. N2-associated haplotype (blue) and CB4856-associated haplotype (red) are plotted separately. Owing to large difference of value, *y* axis is segmented. (**b**) ‘CB4856-derived' SNVs were located at the right end of chromosome V in CB4856, but not in N2. Asterisks indicate CB4856 SNVs. Dashed line indicates genomic position. (**c**) Breakpoint PCR was performed with primer pairs (red arrows in **b**) designed to anneal to the proximal telomere region of chromosome V (VR) and TALT1 in forward (For in **b**) or reverse (Rev in **b**) orientation. CB, CB4856. (**d**) Nucleotide change of WBvar00067712 and WBvar00067717 in internal and telomeric TALT1. N2-associated alleles are blue while CB-associated alleles are red. Nucleotide positions are indicated in **d**.

The mechanism by which TALT regions are utilized at ALT telomeres was elucidated when we analysed the SNVs in this region. We identified two heterozygous SNVs within TALT1 common to both the starting CB4856 strain and the CS survivors. The SNVs were inherited together as two haplotypes, and interestingly, they were unequally represented, with the haplotype normally associated with CB4856 present fivefold higher than the other haplotype, which is commonly associated with the N2 strain (Fig. 5a, Supplementary Fig. 8a–c). This skewing was more pronounced in the CS survivor lines, with a haplotype shift such that haplotype common to CB4856 was highly over-represented compared with the other (Fig. 5a). For CS1, reads containing the N2-associated haplotype were represented at genome-average sequence depth (0.9-fold), while reads containing the CB4856 associated were increased 76-fold above average depth. Similarly, reads from CS2 represented the N2-associated haplotype at average levels (1.2-fold), while reads containing the other were greatly enriched (122-fold) (Fig. 5a). Since TALT1 had been amplified at least 100-fold in these lines (Fig. 2c), we reasoned that the SNVs we identified were actually sequence variation between two discrete loci, with the over-represented CB4856-associated alleles present on the amplified TALT1. To test this we performed PCR across the internal TALT1 on chromosome V, which revealed only the N2-associated alleles represented at genome-average level. Further analysis revealed that the over-represented CB4856-associated allele were located at the right telomere of chromosome V in only the CB4856 strains and CS survivors, but not in the N2 strain (Fig. 5b–d, Supplementary Fig. 5e). We used these variants to distinguish the two locations of TALT1: the internal TALT1 donor (TALT1 D) and the TALT1 reservoir (TALT1 R) at the proximal telomere region.

**Figure 6:**
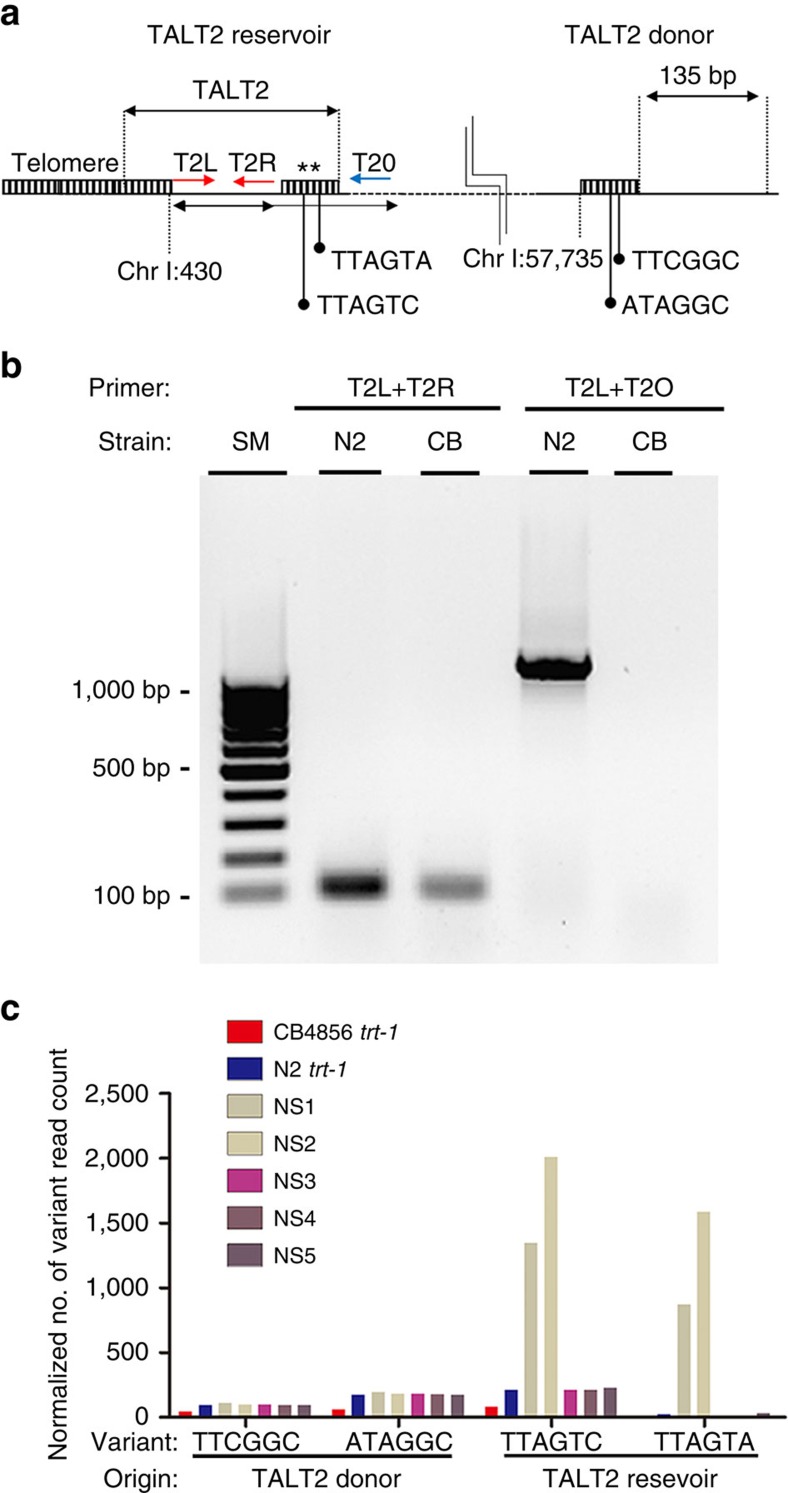
*trans*-Duplication of TALT2 reservoir to other chromosomal ends in N2 backgrounds. (**a**) Diagram of TALT2 in N2 genome. Primers used in **b** are indicated as red arrows. Specific variant repeats (asterisks) of TALT donor and reservoir are indicated. (**b**) TALT2 reservoir is absent in CB4856 wild isolate. PCR amplicons from indicated primers were fractionated by gel electrophoresis and stained with ethidium bromide. CB, CB4856. (**c**) Normalized number of variant read count from WGS data of CB4856 *trt-1*, N2 *trt-1* and all the NS survivors. Variant read count was normalized to total sequencing coverage of each sample.

Similar to the observed TALT1 duplication, we determined that TALT2 also originated from an endogenous locus (TALT2 D) close to the left arm of chromosome I and had duplicated adjacent to the telomere of chromosome I (TALT2 R) (Fig. 6a, Supplementary Fig. 8d). Interestingly, the TALT2 R locus is undetectable in the CB4856 genome (Fig. 6b and Supplementary Figs 5f and 8d), suggesting that CB4856 and N2 have evolved different TALT reservoirs for ALT. Using variant repeats of TALT2 R and TALT2 D, we were able to identify both locations and found that only TALT2 R sequences were over-represented in NS survivors, suggesting that the TALT2 R was used as template for ALT (Fig. 6c and Supplementary Fig. 9a,b).

## Discussion

**Figure 7:**
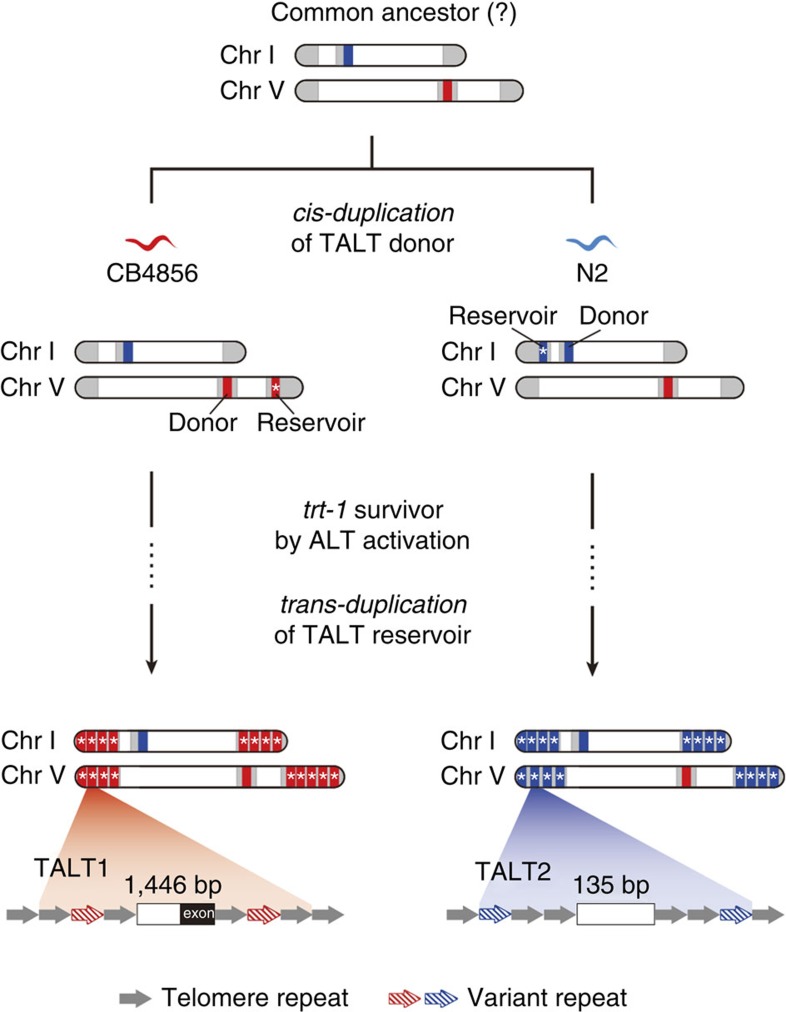
A model for TALT-mediated ALT. Internal genomic regions with ITS are used as template of ALT in *C. elegans* ALT survivors. The model illustrates that after divergence from the hypothetical common ancestor, *cis*-duplication of TALT donor into telomere already occurred independently in nature. After telomeric crisis, the TALT reservoir at a proximal telomere region is used as a template for telomere maintenance.

Based on these observations, we propose a two-step model for TALT utilization in ALT (Fig. 7). The first step involves a *cis*-duplication of a TALT donor to a chromosomal end to create a TALT reservoir before ALT, and the second involves a *trans*-duplication of TALT R to most chromosomes via the ALT pathway. We have demonstrated that this first step event has occurred in wild strains, with TALT from a specific chromosomal ‘donor' region being duplicated in *cis* to the end of the same chromosome. This telomere-adjacent copy (TALT R) acts as a reservoir, so when crisis occurs in individuals, rare survivors recruit the TALT R sequences to all chromosomal ends (*trans*-duplication), stabilizing their chromosomes.

ALT survivors in this study are similar to type I survivors of *Saccharomyces cerevisiae*, in which Y′ elements located to subtelomere are used for telomere lengthening^12^. In humans, it has been shown that a plasmid tag (Tel) integrated within a single telomere duplicated to other telomeres, while a plasmid tag (Subtel) integrated immediately proximal to telomeric DNA did not^13^. While TALT regions are proximal to the telomeric DNA, since they are flanked by an ITS and the telomere, they are more analogous to the plasmid ‘Tel' sequences integrated within the telomeres. Therefore, it is possible that the duplication of regions adjacent to the telomere is an evolutionarily conserved mechanism. Consistent with this, subtelomeric regions in humans rapidly obtained unique sequences during primate evolution^14^,^15^. Likewise, we observed in *C. elegans* that a sequence near the proximal telomere, TALT2, appears to be rapidly changing, as it has been independently duplicated in multiple wild isolates (Supplementary Fig. 10a). We therefore propose that a rapidly evolving proximal telomere region retains highly replicative potential so that it can be preferentially selected as a template for ALT at telomere crisis. Bioinformatic analysis identified more TALT-like sequences in the *C. elegans* reference genome, and it would be of interest to examine the wild isolates that lack TALT1 and TALT2 to assess whether they utilize other TALT-like sequences for ALT (Supplementary Fig. 10b and Supplementary Table 3).

Although we have shown for the first time that internal genomic regions can integrate into the telomeres, resulting in elongation and maintenance of telomeric ends in the absence of functional telomerase, the mechanism by which internal TALT regions are recruited and replicated to the telomeric ends by *cis*- or *trans*-duplication still remains unknown. Unfortunately, we were unable to identify a single factor that suppressed the ALT phenotype in our survivors using a candidate RNAi approach (Supplementary Table 4). Furthermore, while C-circle formation is a well-known marker of human ALT^16^, we did not observe any increase in C-circle formation in our survivors (Supplementary Fig. 11), suggesting that a different mechanism is involved. It is possible that TALT-mediated recombination and unequal sister chromatid exchange plays a role in the duplication to telomeres on different chromosomes^13^ and expansions within telomere tracts from the same chromosome^17^. Without knowing TALT-binding proteins at this moment, it is also difficult to explain how TALT sequences are involved in the capping of chromosomal ends. It is possible that telomere repeats in the TALTs normally function for telomere capping and that telomere variants or non-telomeric sequences found in TALTs may recruit *trans*-acting proteins that do not interfere telomere capping function completely while simultaneously making the telomeres recombinogenic by their protein interaction. This situation could be similar to the proposed ‘intermediate-state telomeres' of ALT cell lines in which the ALT telomeres block chromosome fusions, but not the DNA damage response that can induce recombination^11^. It is also possible that TALT sequences recruit proteins involved in heterochromatinization.

Our discovery of TALT may also help understanding of the mechanism of human ALT. As human ALT cells often incorporate variant telomere repeats that are likely to be originated from proximal telomeres^18^,^19^ and AG11395 ALT cell line has a repeat unit of SV40 DNA with a structure similar to TALT^20^,^21^, it is conceivable that human ALT cells also incorporate TALT elements into their telomeres. Therefore, it would be worthwhile to screen human ALT cell lines and tumours to investigate the presence of a TALT-like mechanism involved in telomere elongation in humans. Our results suggest the presence of TALT R in proximal telomeres is an important DNA signature of ALT activation or initiation.

## Methods

### *C. elegans* strains and culture

Worms were cultured at 20 °C under standard culture conditions^22^. The following strains were used in this study: Bristol N2 wild strain, Hawaiian CB4856 wild isolate, *trt-1(ok410)* I (ref. 7). N2 *trt-1(ok410)* was outcrossed with Hawaiian CB4856 wild isolate to produce CB4856 *trt-1(ok410)*. To ensure the ALT was activated in outcrossed progeny, F2 worms were grown at least 20 generations and worms with low fecundity were excluded. To maximize the outcrossing effect, SNVs of all chromosome markers were checked. The *trt-1(ok410)* mutation was confirmed by PCR and WGS (Supplementary Fig. 1c,d). The *trt-1(ok410)* strain was outcrossed with N2 wild type to produce an early generation of N2 *trt-1(ok410)*.

### EMS treatment

Synchronized L4 worms were treated with 50 mM EMS in M9 buffer for 4 h. After 4 h of recovery, treated P0s were allowed to lay eggs for 12 h. F1 worms were isolated by removing P0 worms. Initially ∼100 F1 eggs were transferred to fresh plate, then from the F2 generation onwards, 10–15 worms were transferred manually at every generation.

### Feeding RNA interference

*Escherichia coli* HT115 expressing dsRNA were grown in Luria–Bertani (LB) with 1 mM ampicillin at 37 °C overnight and seeded on to nematode growth media plates containing 1 mM isopropyl-β-D-thiogalactoside and 1 mM ampicillin. At every generation, 10–15 L1 larvae were transferred to fresh RNAi media plates.

### Telomere florescent *in situ* hybridization

As cells are highly dividing in embryo stage in *C. elegans*, it is most plausible condition that ALT is activated. Eggs were isolated by bleaching adult worms. Eggs were fixed in 2% paraformaldehyde. Tubes containing eggs were frozen in liquid nitrogen and thawed in warm water twice to crack the eggs. Eggs were settled on a slide coated with poly-lysine. The slide was washed three times with phosphate-buffered saline containing 0.1% Tween-20 (PBS-T) to remove residual paraformaldehyde. The slide was incubated in acetone and methanol for 5 min each at −20 °C and was then rehydrated in 2 × SSC (0.3 M NaCl, 0.03 M sodium citrate) containing 0.1% Tween-20. The slide was blocked for 1 h with prehybridization solution 3 × SSC, 50% formamide, 10% dextran sulfate, 50 μg ml^−1^ heparin, 100 μg ml^−1^ yeast tRNA, 100 μg ml^−1^ salmon sperm DNA) at 37 °C. PNA-(TTAGGC)_3_ probe was hybridized for 16 h in humid chamber at 37 °C. Slides were washed twice in wash buffer (2 × SSC and 50% formamide) for 15 min at 37 °C. After washing 3 three times with PBS-T, slides were counterstained with 4,6-diamidino-2-phenylindole and mounted with anti-bleaching solution Vectashield (Vector Laboratory). The samples were imaged using a confocal microscope (LSM700, Zeiss).

TALTs probes were labelled with digoxigenin (DIG) and further visualized by antibody staining. After *in situ* hybridization, slides were blocked with PBS-T containing 5% BSA for 1 h at room temperature. Slides were stained with rhodamine-conjugated anti-DIG antibody for 3 h After washing in PBS-T twice, slides were mounted and observed as described above. The telomere signal was quantified using TFL-TELO software (Dr. Peter Lansdorp, Terry Fox Laboratory, Vancouver).

### Telomere Southern blot

For genomic DNA preparation, worms were collected and washed five times in M9 buffer. Worms were lysed in lysis buffer for 8 h (100 μg ml^−1^ proteinase K, 50 mM KCl, 10 mM Tris (pH 8.3), 2.5 mM MgCl_2_, 0.45% NP-40, 0.45% Tween-20 and 1% beta-mercaptoethanol) DNA was extracted using phenol:chloroform extraction and ethanol precipitaion. DNA in TE buffer was treated with RNAse (10μg ml^−1^) for 2h and re-extracted, before being dissolved in TE buffer.

For Southern hybridization, 5 μg of DNA was treated with 1 unit of restriction enzyme and then separated by gel electrophoresis either using standard equipment or pulsed field gel electrophoresis equipment. Gels were blotted by capillary transfer on to the Zeta probe membrane (Bio-Rad) overnight. The membranes were crosslinked using an ultraviolet crosslinker and hybridized with the Southern probe in DIG Easy Hybridization buffer at 42 °C for 16 h. The membrane was then washed twice at room temperature in 2 × SSC, 0.1% SDS and twice at 42 °C 0.2 × SSC, 0.1% SDS. The DIG-labelled probe was detected on an ImageQuant LAS-4000 biomolecular imager (GE Healthcare) using an anti-DIG-AP antibody chemiluminescence detection kit (Roche). The (TTAGGC)_30_ probe was labelled with DIG-UTP by PCR-amplifying telomere sequences cloned in a T-easy vector. Probes for TALT1 and TALT2 were labelled with DIG-UTP using primers targeting unique region in TALTs. Uncropped scans of blots were supplied in Supplementary Fig. 5.

### C-circle assay

Worm genomic DNA was digested with TALT1 non-cutting restriction enzyme mix (*Nhe*I, *Bam*HI, *Dra*I, *Apa*I, *Nde*I, *Xho*I, *Nco*I and *Sac*I 4 units per μg each). DNA was purified by Phase Lock Gel (5 PRIME) and ethanol precipitation. A volume of 10 μl of sample was mixed with 10 μl 0.2 mg ml^−1^ BSA, 0.1% Tween-20, 1 mM each dATP, dGTP, dTTP and dCTP, 1 × phi29 buffer with or without 7.5 units phi polymerase (NEB) and incubated at 30 °C for 8 h then at 65 °C (polymerase inactivation) for 20 min. For dot blotting, samples were diluted with 60 μl 2 × SSC and blotted onto nylon membrane. DNA was crosslinked with ultraviolet on to the membrane and hybridized at the 62 °C with (GCCTAA)_4_-digoxigenin probe in DIG easy hybridization buffer (Roche). C-circle amplified signal was detected by DIG detection kit (Roche) according to the manufacturers' instructions. *pot-1(tm1620)* and *pot-2(tm1400)* were used as positive controls as they are reported to contain elevated levels of C-circles compared with N2 wild type^23^,^24^. For sample loading confirmation, we stripped by 0.2 M NaOH, 2% SDS and re-hybridization with (GCCTAA)_4_-digoxigenin probe (denatured blot).

### BAL 31 exonuclease treatment

A total of 5 μg of DNA was treated with 10 units of BAL 31 at 30 °C in 1 × BAL 31 buffer. Reactions were stopped with the addition of EGTA (25 mM final concentration). DNA was collected by ethanol precipitation and digested with non-cutting restriction enzyme mix (*Nhe*I, *Bam*HI, *Dra*I, *Apa*I, *Nde*I, *Xho*I, *Nco*I and *Sac*I).

### Sequence analysis of telomere–proximal telomere region junctions

To analyse the exact sequence of the junction of telomere and proximal telomere region, PCR products of junctions were sequenced. For PCR reactions, the forward primer was designed against telomere-adjacent DNA to elongate into the telomere and reverse primer was designed from TALT1-specific sequence (the first exon of T26H2.5). CS1 genomic DNA and N2 genomic DNA were used as template. In all, 30 PCR cycles were performed with primers annealing at 60 PC and elongation progressing for 3 min After electrophoresis in 1% agarose gel, the CS1-specific amplicon was gel extracted and sequenced. Primers used for sequencing were the same as those used for PCR reactions.

### Whole-genome sequencing

DNA was fragmented to 300 bp and sequencing libraries were constructed with an average insert size of 430 bp using standard Illumina protocols. Libraries were run on an Illumina Hiseq2000 sequencing platform.

### Variant discovery

To analyse variant induce by EMS and CB4856-associated variant, WS243 version of *C. elegans* reference genome and annotation data were acquired from Wormbase website (www.wormbase.org). For preprocessing the raw sequencing data, Trimmomatics was applied so that illumina adaptor sequences were removed^25^. The 3′ and 5′ ends of low-quality reads were trimmed. Preprocessed reads were then aligned to *C. elegans* reference genome (WS243) with the Burrows-Wheeler Aligner software using default parameters (version 0.7.5a)^26^. Before calling variants, Picard's SortSam and MarkDuplicates and GATK's indel realignment^27^ and base quality score recalibration was applied. For the indel realignment, very stringent parameters were used due to the probability of considerable genetic difference between the reference assembly and survivor lines. From reference annotation (WS243), SNVs and indels sourced from the million mutation project were selected and used in base quality score recalibration. Variant discovery and genotyping were performed across all samples simultaneously using GATK's haplotype caller as per GATK Best Practices, then standard hard filtering was applied with minimum raw depth of coverage of 4.

### TALT1 contig generation

Since TALT1 is amplified from CB4856 allele (TALT1 R) that is absent from genome assembly (based on the N2 genome), we reconstructed TALT1 R region through *de novo* assembly of CB4856 *trt-1* sequencing data using CLCworkbench7 (Qiagen) with default setting. Using BWA, reads of CS1 were collected if only one of the pair was mapped on the reconstructed TALT1 reservoir. Collected reads were used to generate contig sequences, which were then aligned to N2 genome using BLAST.

### Whole-genome sequencing analysis for telomere reads

WGS data were aligned to the ce10/WBcel215 reference assembly using bwa^26^ and converted into bam files using Samtools^28^. Bam files from different worm strains were analysed for reads predicted to derive from within telomeres using previously described software (http://sourceforge.net/projects/motifcounter/)^18^. Briefly, reads that contained at least six canonical telomere repeats (TTAGGC) were extracted and saved into separate files. The number of reads was counted and normalized to total read density to infer telomere length. The saved files were subsequently used to analyse split-pair regions and for variant calling.

### TALT bioinformatic analyses

To identify regions that were associated with the telomere, files containing telomeric reads were used to extract their associated paired ends. Reads in which both pairs were telomeric were excluded, leaving only reads in which one pair mapped to a genomic location. In addition to subtelomeric regions and loci flanking interstitial telomeres, the majority of the reads mapped to TALT1 in the case of CS1 and CS2, and TALT2 in the case of NS1 and NS2.

Average genomic coverage across the genome was calculated using genomecov from the BedTools package^29^, after the removal of duplicates and low mapping quality (*q*<20) reads in Samtools. The read depth was assessed at every nucleotide in the genome and normalized by dividing by the average coverage to give a fold-change metric for read depth. The fold-change read depth from the parental strains was subtracted from the ALT survivors to account for strain-specific variations across the genome. Line plots of genome-wide and locus-specific read depth changes were created in R. Putative additional TALT regions were identified by analysing the *C. elegans* assembly with the Homer scanMotifGenomeWide function^30^ for (TTAGGC)_7_ with up to three mismatches to find all genomic ITS elements. The distance between each ITS was calculated, and data was extracted if this distance was between 100 and 2,000 bp. All candidate regions were manually confirmed, the 5′ and 3′ ITS length recorded, and any genes noted.

### Telomere variant calling

Telomere variants were analysed from files containing telomere reads. The number of each variant type was counted within this subset of reads from each library using the programme motif_counter^18^. The presence of each variant repeat was assessed within this subset of reads, and their frequency was calculated and used to generate pie charts.

### Measurement of TALT copy numbers

TALT copy numbers were measured from worm total genomic DNA by using quantitative real time PCR. We calculated TALT copy number normalized by a single-copy gene, *act-1*. Each reaction performed on the iCycler iQ5 (Bio-Rad) using following thermal profile: 95 °C for 3 min; 40 cycles of 95 °C for 20 s, 60 °C anneal for 20 s, 72 °C extend for 30  s along with 81 cycles of melting curve from 60 to 95 °C. The reaction The reaction components are as follows : 10 μl 2 × SYBR Green mix (Bio-Rad), 1 μl each of 10 μM forward and reverse primers (Supplementary Table 5), 7 μl sterile water and 1 μl total genomic DNA (100 ng μl^−1^) in 20 μl reaction.

### mRNA sequencing analysis

To align the preprocessed RNA-seq reads to reference genome and estimate the expression level of transcripts, we followed the Tuxedo pipeline (Tophat2 and Cufflinks pipeline)^31^. First, Tophat2 mapped RNA-seq reads to the *C. elegans* genome using given transcript annotation (WS243) (ref. 32). Next, using cuffquant and cuffnorm software in Cufflinks package^33^, we estimate the abundance of known transcripts in normalized RPKM across all samples.

### Gene set enrichment analysis

The gene ontology annotation data for *C. elegans* were downloaded from the Wormbase website (www.wormbase.org). Gene sets that change on irradiation was obtained from ref. 10. For all pairs of ALT samples and a wild type, Gene Set Enrichment Analysis was performed using the gene set data obtained above^34^.

## Additional information

**Accession codes:** RNA-seq data of ALT survivors are available in the Sequence Read Archive at the National Center for Biotechnology Information under the accession code SRA278191.

**How to cite this article:** Seo, B. *et al*. Telomere maintenance through recruitment of internal genomic regions. *Nat. Commun.* 6:8189 doi: 10.1038/ncomms9189 (2015).

## Supplementary Material

Supplementary Information Supplementary Figures 1-11, Supplementary Tables 1-5
