# Supplementary material for: Telomere maintenance through recruitment of internal genomic regions
Source: Nat Commun. 2015 Sep 18;6:8189. doi: 10.1038/ncomms9189 (PMC4595603; doi:10.1038/ncomms9189)
Supplement: Supplementary Information — Supplementary Figures 1-11, Supplementary Tables 1-5 [file ncomms9189-s1.pdf]

Supplementary Figures

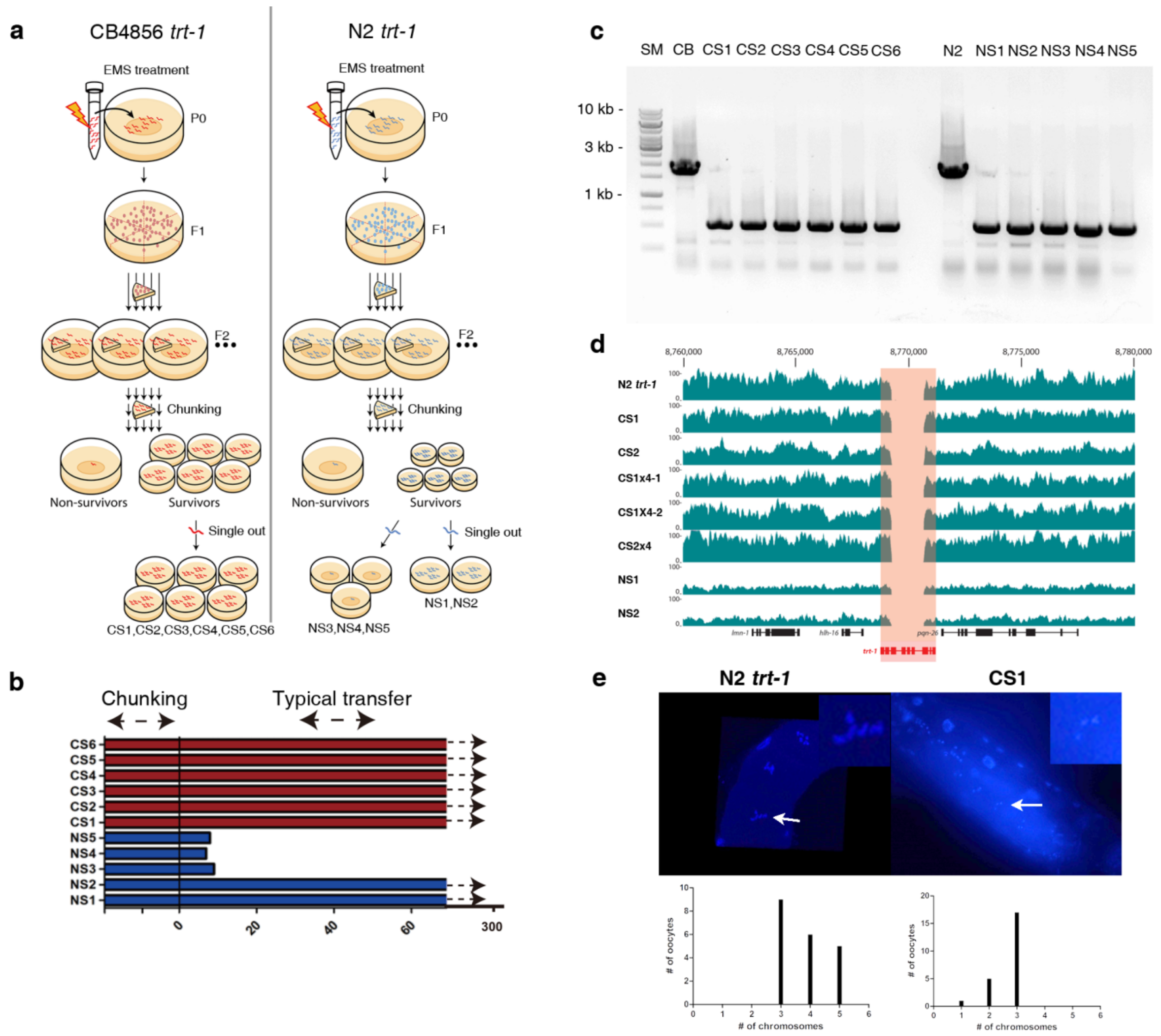

Supplementary Figure 1. A schematic diagram of the isolation protocol for ALT survivors (a) For CS survivors, worms were separated to 80 plates after EMS treatment. After 8 generations, 6 resulting survivors (CS1-CS6) were maintained by transfer of small number of worms each generation. For N2 survivors, worms were separated to 200 plates after EMS treatment. 5 survivors (NS1 – NS5) were maintained by large chunking, two of which (NS1 and NS2) were subsequently maintained by transfer of small number of worms each generation. **(b)** Survival graph of ALT survivors. NS3, NS4 and NS5 reached sterility within 10 generation (y-axis) by transferring 10-15 larvae. **(c)** PCR amplification of the *trt-1* deletion allele. The *trt-1(ok410)* deletion mutation results in PCR amplicon shorter than wild-type control. CB, CB4856. **(d)** Coverage plot of whole genome sequencing reads around the *trt-1* locus confirms the deletion. **(e)** Histogram of chromosome numbers of N2 *trt-1* and CS1 in diakinesis of the germline. Top right corner shows the magnified images of nuclei indicated by arrows. Total n= 20, 21 respectively.

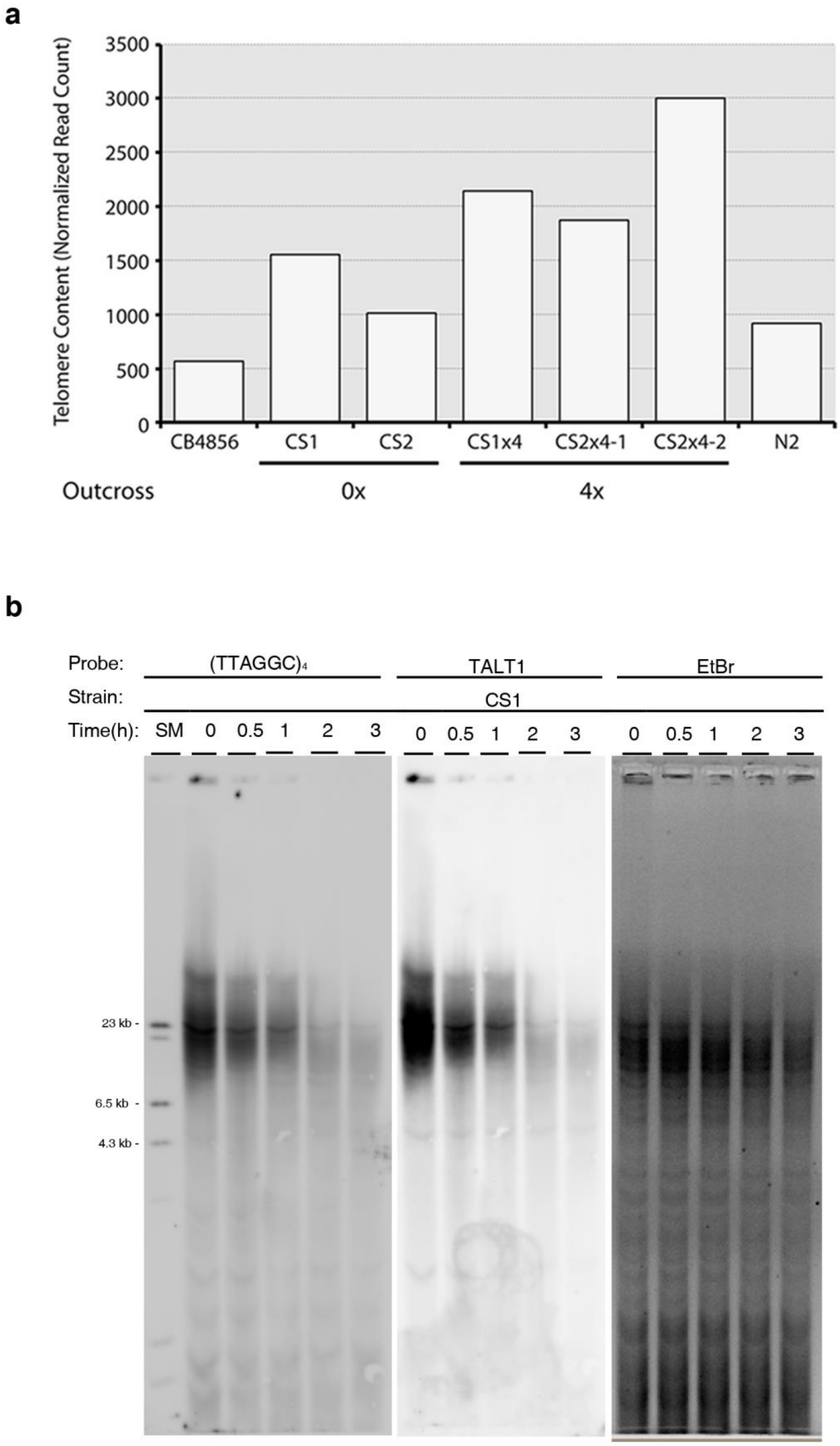

Supplementary Figure 2. Telomere length was increased in CS survivor at the chromosome ends (a) Normalized count of reads containing at least 6 telomere repeats in CB4856 *trt-1,* CS1, CS2 and outcrossed CS1 and CS2 worms. **(b)** BAL 31 exonuclease assay of CS1. Genomic DNA was treated by BAL 31 exonuclease prior to digestion with TALT1 non-cutting restriction enzyme mix. Digested DNA was then analyzed by Southern blot.

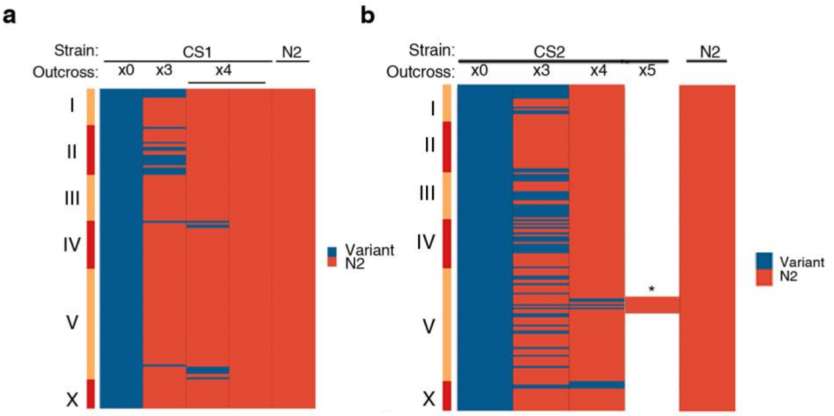

c

Strain	ORF name	Gene name	Status	Predicted function	Southern lane
CS1x4-1	2L52.1		deletion	ZF TF	1
CS1x4-1	2RSSE.1		deletion	RhoGAP domain	9
CS1x4-1	2RSSE.2		deletion	RhoGAP domain	10
CS1x4-1	Y67D2.6		deletion	RNA helicase	11
CS1x4-1	Y38C1AB.4	frm-5.2	deletion	FERM domain	13
CS1x4-1	Y38C1AB.8	frm-5.1	deletion	FERM domain	13
CS1x4-2	Y66A7A.5	ceh-91	deletion	reproduction 관련	4
CS1x4-2	Y66A7A.4		deletion	unknown	3
CS1x4-2	Y38C1AB.4	frm-5.2	deletion	FERM domain	13
CS1x4-2	Y38C1AB.8	frm-5.1	deletion	FERM domain	13
CS2x4	Y74C9A.3	homt-1	deletion	Hydroxyindole-O-MethylTransferase homolog	7
CS2x4	W02B12.11		deletion	carbohydroxy	2
CS2x4	K04F1.15	alh-2	deletion	dehydrogenase	5
CS2x4	B0213.10	cyp-34A5	deletion	cytochrom P450	8
CS2x4	Y38A10A.7		deletion	unknown	12
CS2x4	cTel55X.1		deletion	unknown	6

d

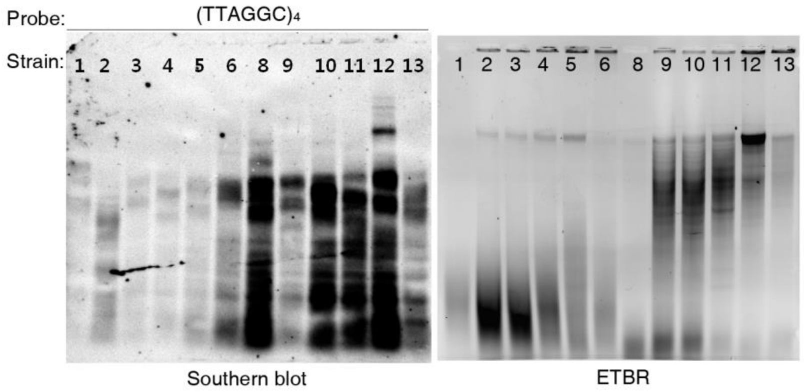

Supplementary Figure 3. No single mutation was responsible for inducing ALT (a) Mutational heatmap of CS1 across the genome. None of the variants that were present in the initial isolates were maintained after outcrosses. **(b)** Mutational heatmap of CS2 across the genome. CS2 survivor was outcrossed with N2 trt-1 by five rounds. Remaining variant on X chromosome was affecting pseudogene (Y35H6.3). Asterisk indicates N2 genetic background confirmed by snip-SNPs mapping using pKP5113, pKP5114 and pKP5116 SNPs. **(c)** Deletions detected by CGH were not responsible for inducing ALT in N2 *trt-1*. List of candidate genes that have deletions in exons. Yellow indicates common candidates of CS1X4-1 and CS1X4-2. **(d)** Candidate genes were inhibited by feeding RNAi to N2 *trt-1* mutant for 10 generations. There was no increase in trans-generational lifespan of N2 *trt-1* after treating with RNAi, and no telomere lengthening was detected. Telomere lengths were measured by Southern blot.

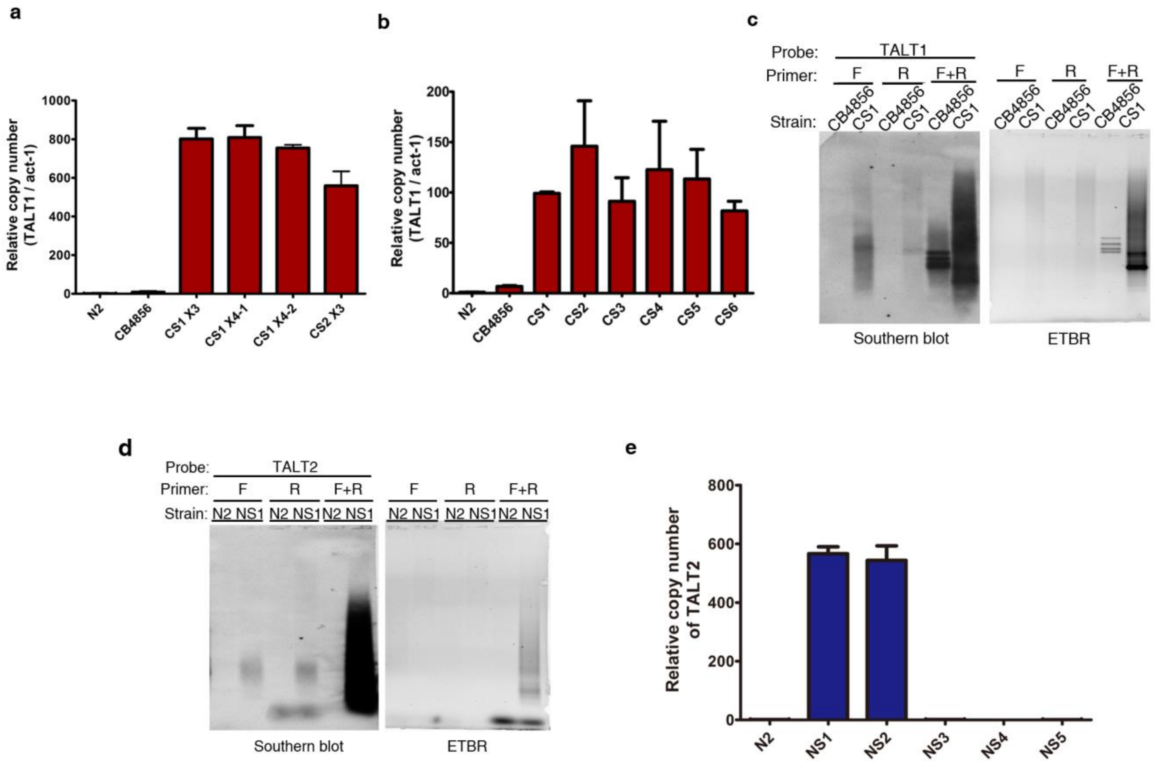

Supplementary Figure 4. ALT survivors have increased copy number of TALT (a) Copy number measurement of TALT1 by quantitative PCR (qPCR). After outcrosses to N2, TALT1 copy number increased in outcrossed CS survivors. Copy number was normalized to the *act-1* gene. $n = 3$. Bar is ± S.D. **(b)** Copy number measurement of TALT1 by qPCR in all CS survivors. $n = 3$. Bar is ± S.D. **(c)** Amplified TALT1 exhibits tandem repeat patterns. PCR was done with single primer, either forward or reverse, to detect head-to-head or tail-to-tail orientation. To detect tandem repeat, both forward and reverse primer was included in PCR reaction. Amplicons were separated by gel electrophoresis and hybridized with TALT1 probe. **(d)** Amplified TALT2 exhibits tandem repeat patterns. **(e)** Copy number measurement by qPCR using a TALT2-specific primer. Both stable NS survivors (NS1, NS2) have increased level of TALT2. Copy number was normalized to the *act-1* gene. $n = 3$. Bar is ± S.D.

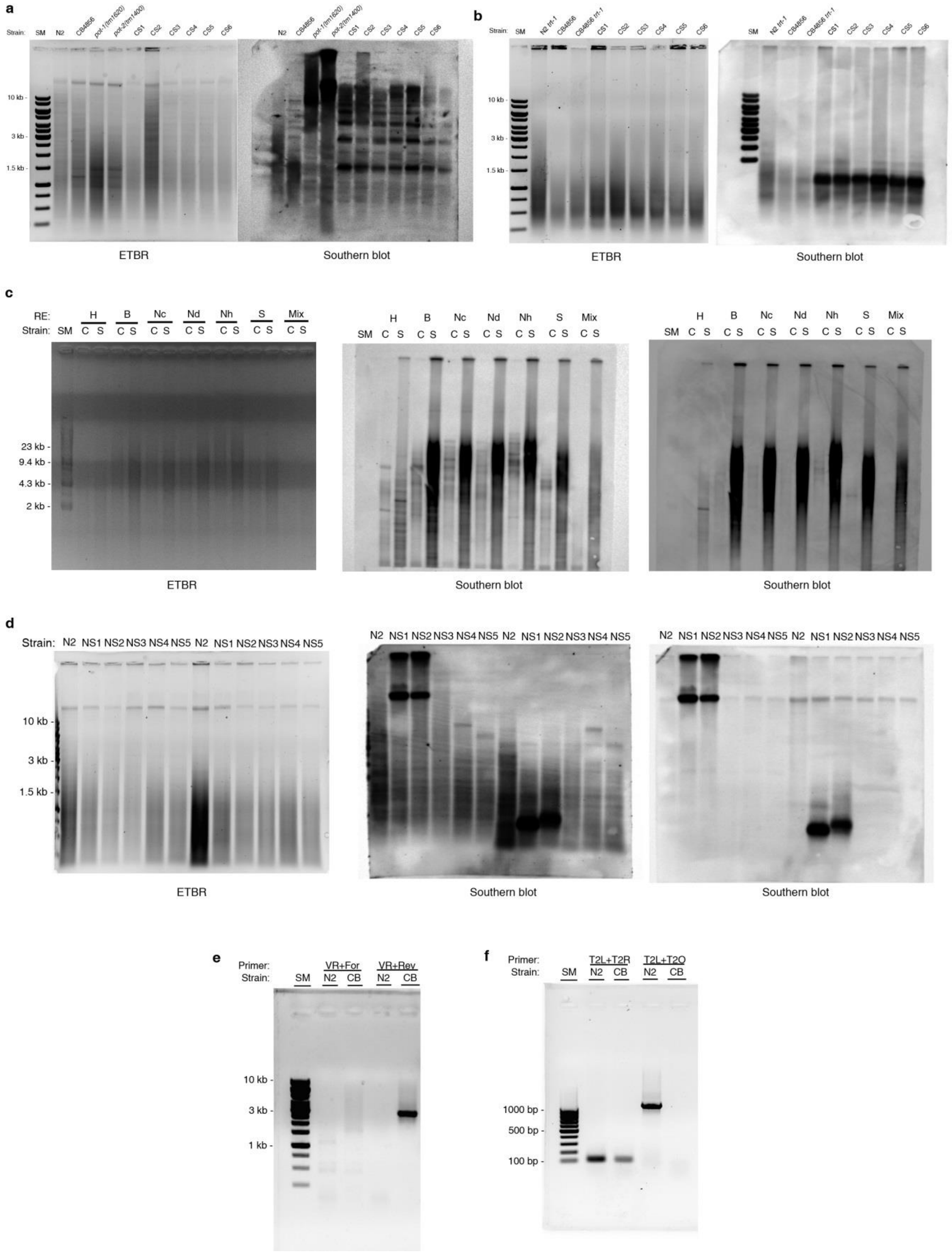

Supplementary Figure 5. **Ethidium bromide stained gel image (a)** Ethidium bromide stained gel image and uncropped southern blot of Fig. 1c **(b)** Ethidium bromide stained gel image and uncropped southern blot of Fig. 1d **(c)** Ethidium bromide stained gel image and uncropped southern blot of Fig. 3d **(d)** Ethidium bromide stained gel image and uncropped southern blot of Fig. 4a **(e)** Uncropped gel image of Fig. 5c **(f)** Uncropped gel image of Fig. 6b

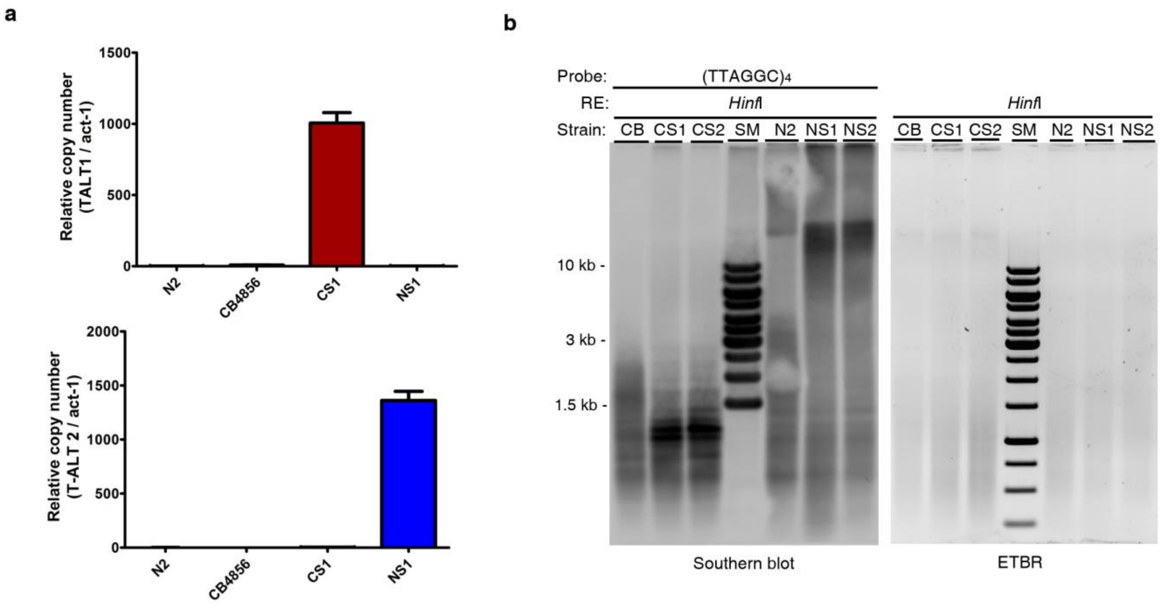

c

	TALT1	TALT2
Background	CB4856	N2
Origin	Chromosome V	Chromosome I
Telomere variant	TCAGGC	TTAGTC, TTAGTA
Unique sequence length	1446	135

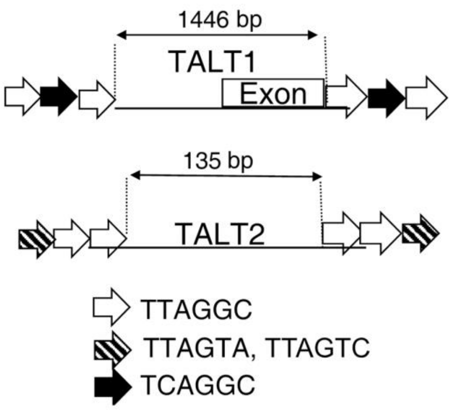

Supplementary Figure 6. **ALT survivors utilize different TALT loci in a strain-dependent manner (a)** Copy number of TALT1 specifically increased in CS1, while copy number of TALT2 specifically increased in NS1, assessed by qPCR and normalized with *act-1*. *n* = 3. Bar is ± S.D. **(b)** CS and NS survivor show different TRF pattern. **(c)** A schematic diagram of TALT elements. The table summarizes the characteristics of TALT1 and TALT2. The right panel shows the structure of TALT elements.

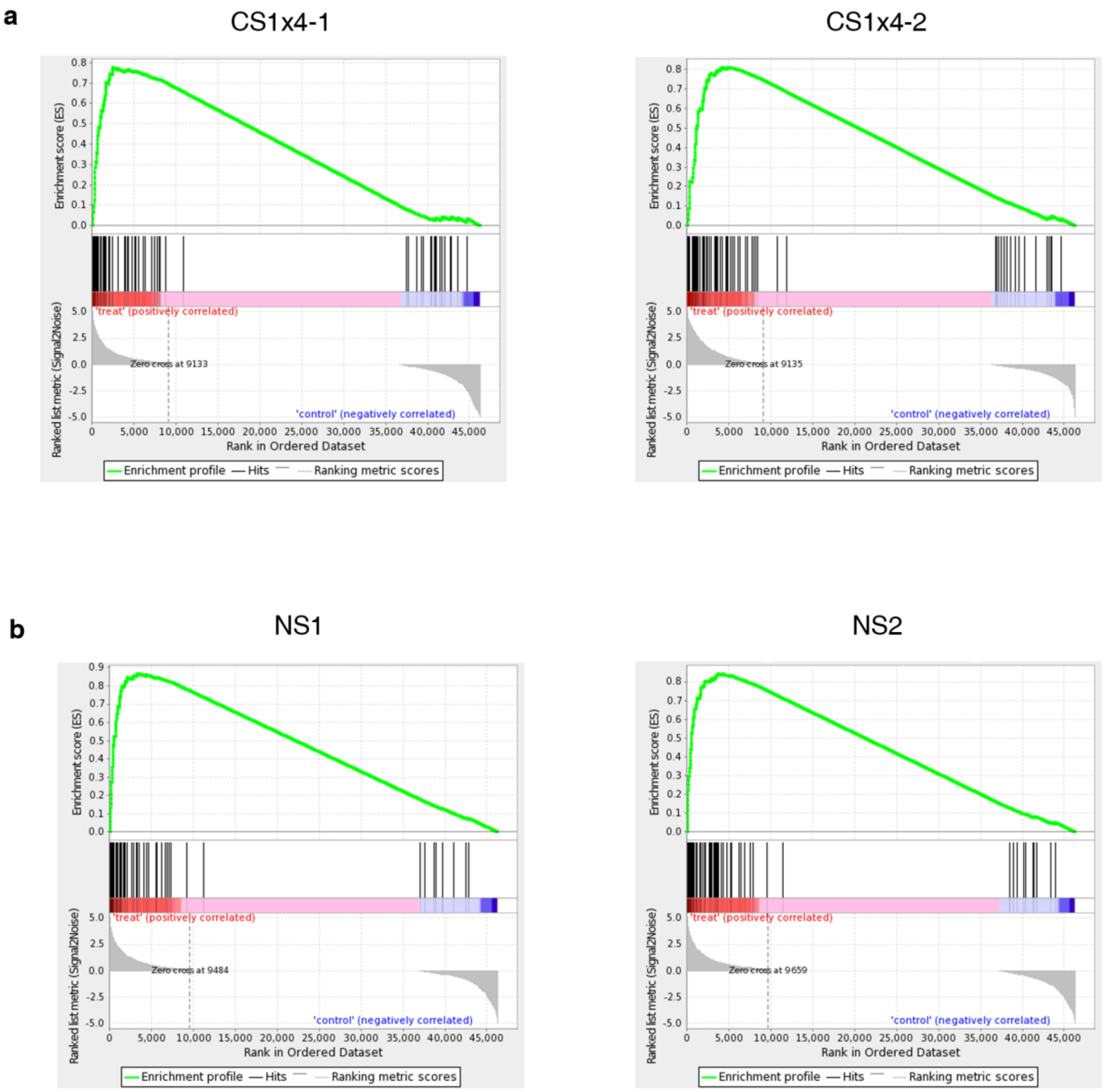

Supplementary Figure 7. Gamma-ray responsive gene set was enriched in all ALT survivors (a) GSEA results of CB4856 survivors. Gamma-ray responsive gene sets are enriched in CS1x4-1 and Cs1x4-2 survivors. This gene set significantly enriched at FDR *q*-value < 0.005 in both strains. **(b)** GSEA results of N2 survivors. Gamma-ray responsive gene sets are enriched in NS1 and NS2 survivors. This gene set significantly enriched at FDR *q*-value < 0.005 in both strains.

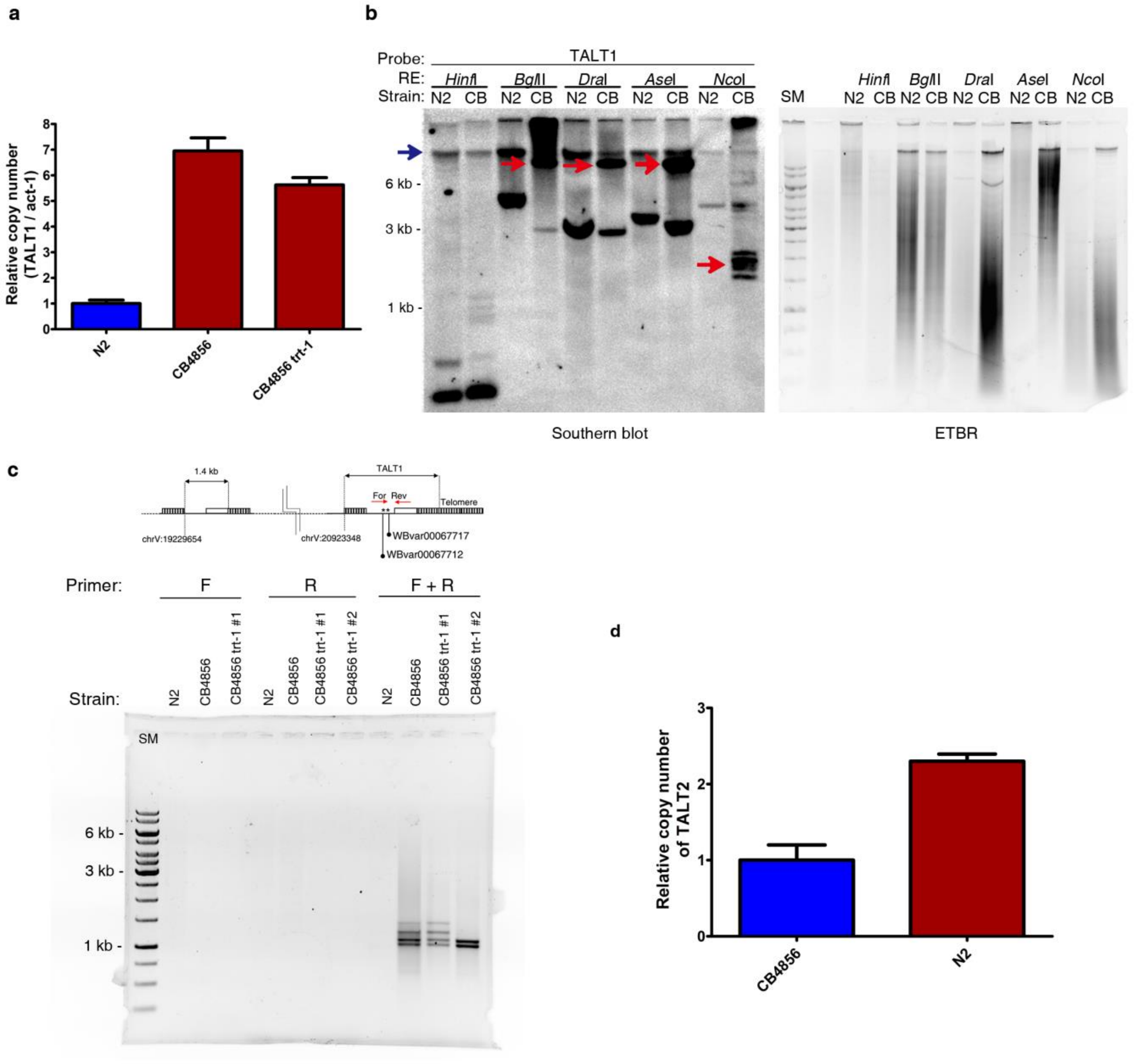

Supplementary Figure 8. The TALT locus is duplicated in both wild strains (a) Copy number of TALT1 DNA is higher in CB4856 than N2. Quantitative PCR (qPCR) using TALT1 specific primers showed 8 folds increase in CB4856 than N2. Fold changes are normalized with single copy gene, *act-1*. *n* = 3. Bar is ± S.D. **(b)** CB4856 TALT1 shows distinct restriction fragments length polymorphism (RFLP) patterns compared to N2. Fragments that do not exist in N2 was detected in CB4856. Using enzymes that did not cut TALT1, CB4856 TALT1 had other large products that were unexpected from sequence information. The blot was probed with TALT1. Blue arrow indicate incompletely digested fragment of genomic DNA. Red arrows indicate another copy of T26H2.5 locus. **(c)** TALT1 elements were tandemly duplicated in CB4856. F and R primers were specific for the CB4856-derived allele. TALT1 amplification was detected by southern blot with a TALT1 probe. Blue arrow: uncut DNA. Red arrow: CB4856-specific restriction fragment. **(d)** Copy number of TALT2 specifically increased in N2 assessed by qPCR and normalized with *act-1*. *n* = 3. Bar is ± S.D.

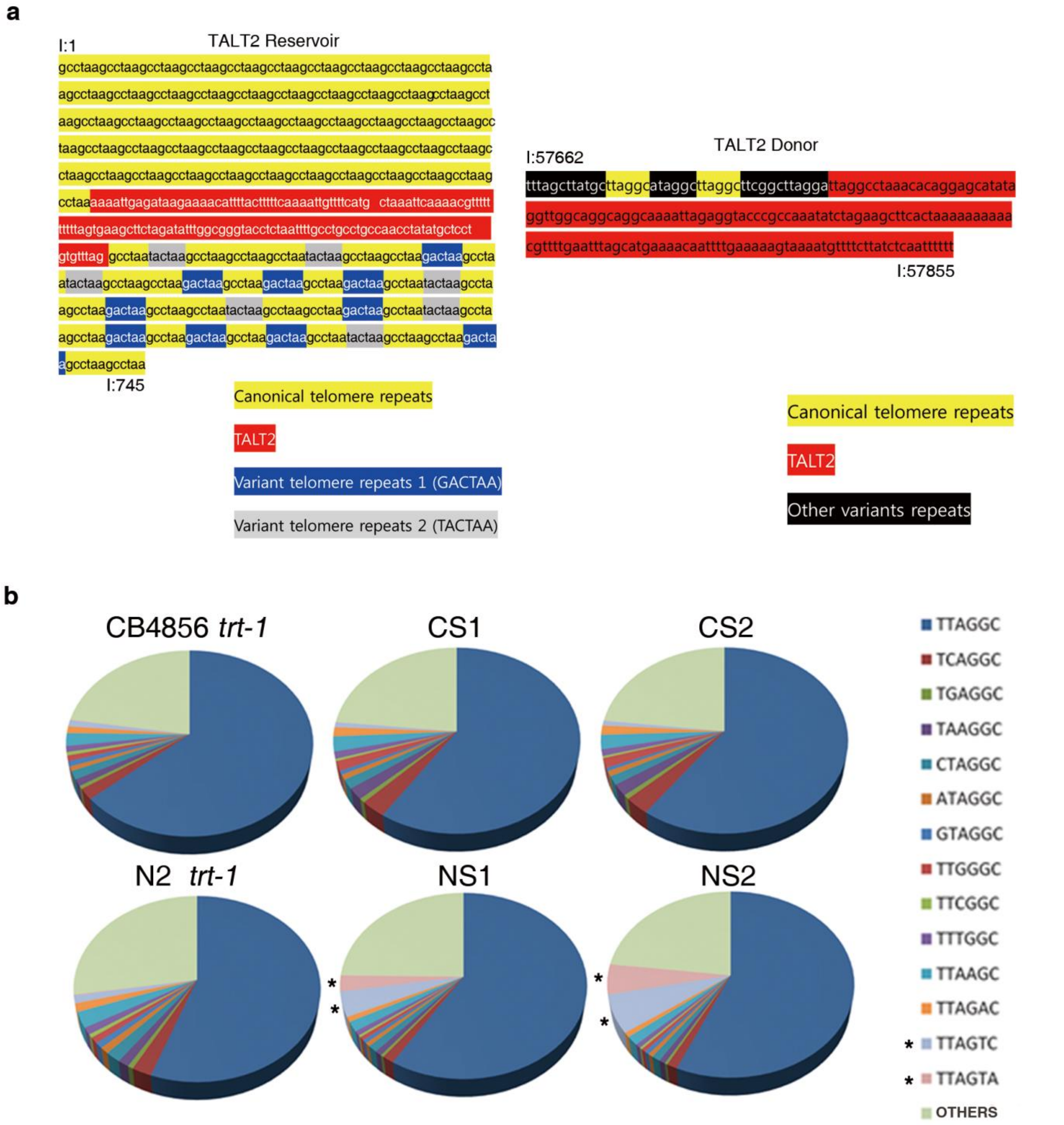

Supplementary Figure 9. TALT2 reservoir is amplified in NS survivor (a) Sequence information of TALT2 donor and reservoir site. Red: TALT2, Yellow: canonical telomere repeats, Blue: variants telomere repeats 1 (GACTAA), Gray: variant telomere repeat 2, Black: other variants repeats. **(b)** Differences in variant telomere repeat usage were detected in different survivors. Telomere reads were defined as sequence reads with at least 6 non-consecutive TNAGGC repeats. The frequency of each variant within these reads was calculated and used to create pie charts. Asterisk (*) represents increased variants repeats in NS survivors.

a

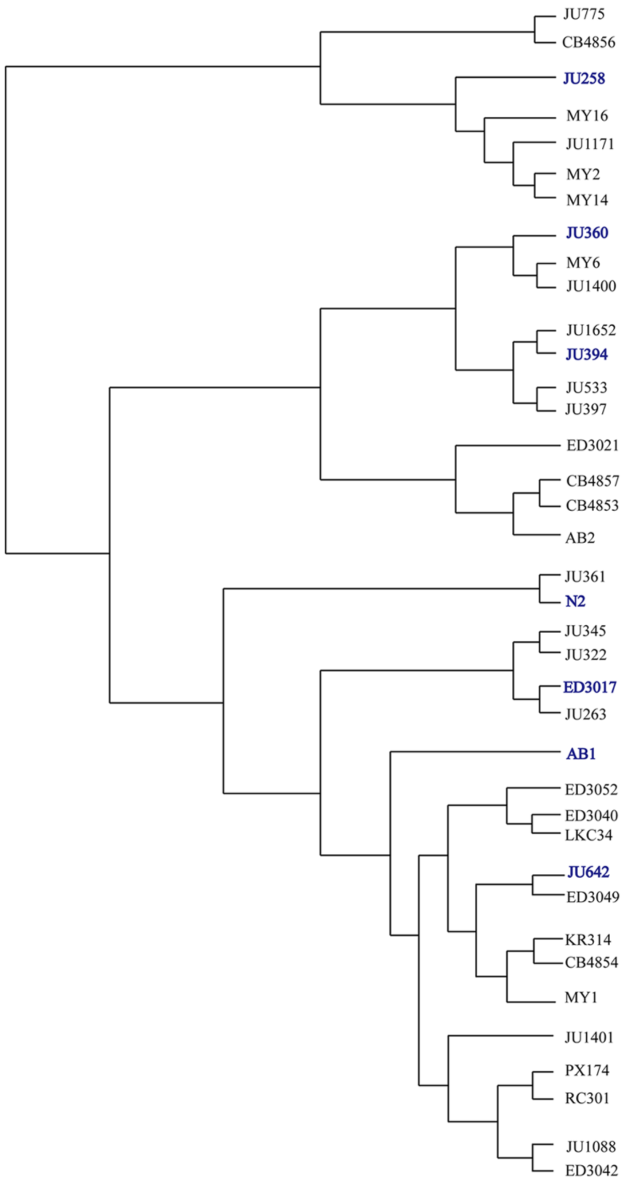

b

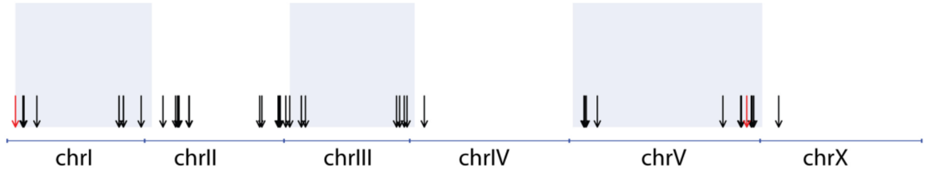

Supplementary Figure 10. *Cis*-duplication of TALT to proximal telomere has occurred independently multiple times (a) The figure shows the phylogenic tree of 38 wild strains whose genomes were fully sequenced (simplified from ref. 8). The blue color-coded strains contain N2 type TALT on chromosome I. **(b)** Potential TALT candidates. Genomic locations of potential TALT are marked by arrows. Red arrows indicate TALTs identified in this study. Potential TALT elements are defined as a region between 100-2,000 bp flanked by ITS.

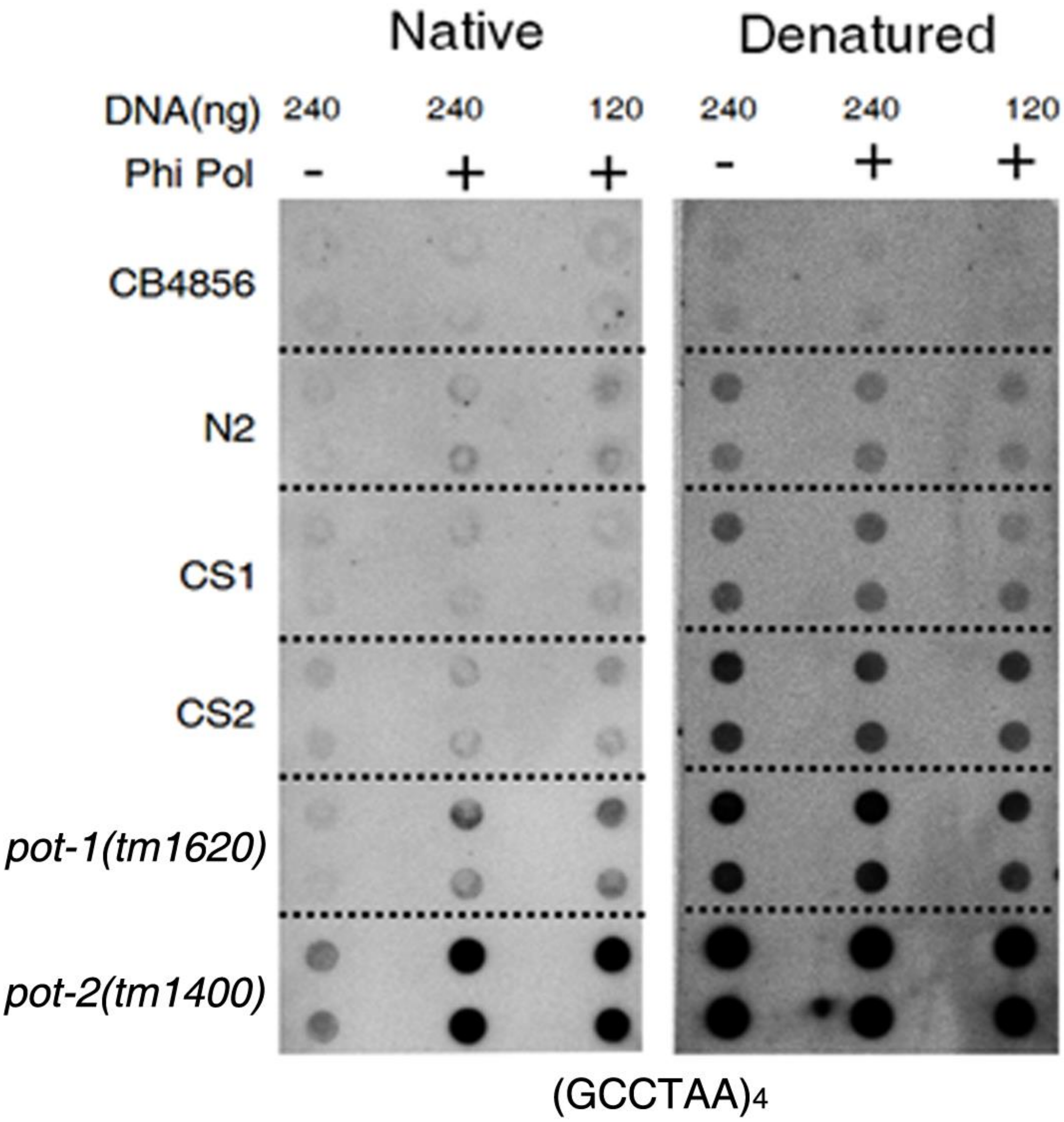

Supplementary Figure 11. C-circle was not detected in ALT survivors Left panel shows native condition that detects only single strand DNA amplified by phi polymerase. As a negative control, the same blot was denatured and double stranded template DNA was detected in right panel. *pot-1(tm1620)* and *pot-2(tm1400)* was used as positive control which is known to have C-circle.

Supplementary Tables

Supplementary Table 1. List of contigs constructed using telomere read-containing reads

Chr.	Location	Sequence*	Confirmed by PCR
**IR**	15071743<br>~<br>15072072	GGCACTAGTACTATGCGCCGCGAGACACACACTACCATCACCAACACAGCCCC<br>CTGAGACATCACGTCTCAGACCAACTGTCACCCCCCCTGACACATATAGGAGTG<br>TGCGGGGAGGGAGTATTATAAAAACCACGGAAAGCCGGCAGTGTGCAATTTGA<br>GAGACGGCAGACAACGCGCGCACTACCCCCCACCACACCGACCCCCTTCCTCG<br>AGACACCGTTTTTCTCAGCCGTCTCTCTGGAGACCTCGGCCGCTTGCACAAAAT<br>TTTCTATCATTTTGAGACTTTGGTGGTGTGTCCCCCTCTCCACCAGCACTGCGGC<br>TGCTGCCTACAGCAGAGTGGAGAATACGTGGGGGATAAGTAAGATTTTTCTAT<br>TTTTCATAAGCCTA	O
**IIR**	15255618<br>~<br>15256023	CGAACTTCGAGAACAAACTTAGGCTTATTACGGCCGACGATTTCAAATTAAAA<br>AAAAAACTGAAAAATTCAGGGCGAATTTGTTAATATTTTTATGTTAGCAACCAT<br>GAGAGACGCAGAGAGAGAGACAGAAAGATGAAAAAGGGCGCTTGCGGACGTC<br>GTCGGGTAGGGTGCCTGAAGTCGATGCGAAGAGATGGGAGTGGAGAGAGTGT<br>GGTCACCATGAGAGACGCAGACATGTACACACATACTCTACGTCTCTCTGAGA<br>AGAGCCGCTGTGCTACTACTATGGGTGCAATAGTATGGAGAAGATGAGAGATG<br>AACCTTTTTAGATTAGAATATTTTGAAAACGTGGGATTCGTTTTTAAATAGATT<br>TTACTGAAATATTGCTGCGAAATTTCCATCAATTTTTTTTCTTTTTTGGAAAAAC<br>ACAAAAATCAAACTACGGTATAAGGTTGCTAACATAAAAATATTAACAAATTC<br>GCCCTGAATTTTTCAGTTTTTTTTTTAATTTGAAATCGTCGGCCGTAATAAGCCT<br>AAG	O
**IIIL**	119 ~ 332	ATTTATCGATTCTTATCGATTTTCTTCTCTTTCCGAACCTTTTCGGAATCAAAAA<br>GCCGTGAATCCATAGATTCCGTGCTTTCTCAGACTTTTCGAGGCCTAATTTTGG<br>TCGAAAAGCCCGATTTTATTTTTATCTGATTTCCGACTCTTTTCGGACTCAAAAA<br>GTTATAAATCCTTGAAATTTCATAGGATTTCGCGCTT**TTAGGCTTAGGCT**	O
**VR**	20923658<br>~<br>20923995	AACGGACAAAAACTTTAAAAACCTCCTGTAAAAGAACCTGGGCTGAGCAAAA<br>GCACATGAAAATTTTTTGAATAAAATATCATACTTCATACACCATATACCCATA<br>AAGTAATAAGAATCTAATGGCAACAGAGGATACTGGTAAGAATCTAATGGCGA<br>CAGAGGATACTGGGAATTTTTATCTGTAACTTATTGGCATCCTTAATAGCATAA<br>AGGTTTATTGAATGTTTTCAAGCTATTTTGAAAATTCTTTAATTCCGCGAAGTTT<br>CTTGATTTCTAAATTACTGAACAAAAACCAGCTACTTGCGTAAGGTACTGAACC<br>ACTTTTCTGGGGATTTGC**TTAGGCTTAGGCTTAGGCTTAGGCTTAGGCTTAG**<br>**GCTTAGGCTTAGGCTTAGGCTAAGGCTTAGGCACAGGCTCAGGCTTAG**	X
**XR**	17718698<br>~<br>17718815	CCAAAGAACAAAAAAAGAAATTAAAATATTTATTTTGCTGTGGTTTTTGATGTG<br>TGTTTTTTATAATGATTTTTGATGTGACCAATTGTACTTTTCCTTTAAATGAAAT<br>GTAATCTTAAATGTATTTCCGACGAATTCGAGGCCTGAAAAGTGTGACGCCATT<br>CGTATTTGATTTGGGTTTACTATCGAATAATGAGAATTTTCAGGC**TTAGGCTTA**<br>G	X

* Red, TALT sequence; Yellow, Telomere repeat

Supplementary Table 2. The list of junctions between chromosome end and TALT in ALT survivors

Strain	Chr.	Sequence*
**CS1**	**IR**	TCCAACCCGAATGATGGAGATTTTTTAGGTAGCTTTTTTTAGACCAATAGCAAATTCATAGT<br>CCGACTAGCCTATAGACTTGGGCTTAGGCTTAGGCTCAGGCTCAGGCTTAGACTTATGCTA<br>AGGCTTATGCTTTTAATTAGGCTTAGGCTTAGGCTAAGGCATTCACTTAGGCTTAGGTTTAG<br>GCTTAGGCTTCGGTTCGAGCTGAGGCTCAGACTTAAGCTTAGGCTCAGGCTTAGGCTTAGG<br>CTTAGACTTTGGATTAAGCTTAGGCTTTGGTATAGGCTTAGGCTTAGGTTTAGGCTTAGGCT<br>TGGGCTTGGGTTGGGCTTGGGCTTGGGTTGGGCTCAGGCTTAGGCTTAGGCTTAGGCTTAG<br>GCTTAGGCTTAGGCTTAGGCTTAGGCTTAGGCTTAGGCTTAGGCTTAGGCTTAGGCTTAGG<br>CTTAGGCTTAGGCTTAGGCTTAGGCTTAGGCTTAGGCTTAGGCTTAGGCTTAGGCTTAGGCT<br>TAGGCTTAGGCTTAGGCTTATGAAAAATAGAAAAATCTTACTTATCCCCCACGTATTCTCC<br>ACTCTGCTGTAGGCAGCAGCCGCAGTGCTGGTGGAGAGGGGGACACACCACCAAAGTCTC<br>AAAATGATAGAAAATTTTGTGCAAGCGGCCGAGGTCTCCAGAGAGACGGCTGAGAAAAAC<br>GGTGTCTCGAGGAACGGGGTCGGTGTGGTGGGGGGTAGTGCGCGCGTTGTCTGCCGTCTCT<br>CAAATTGCACACTGCCGGCTTTCCG
**CS1**	**IIR**	GTCTTATCACCAAACTCTGTATAAAACGAAGGATGGGATGGAACTGAAGCATAAAGTTTCT<br>ACTAACTTTCTCCAATTTCTCCAATTCCATCAATGGAAGCCCGTGAGCCCTCAAGTCTTGCC<br>GAGGAGCTGGAGCTGGTGCCCCCCGAGCTTCGATCGAACTTCGAGAACAAACTTAGGCTTA<br>TTACGGCCGACGATTTCAAATTAAAAAAAAAAACTGAAAAATTCAGGGCGAATTTGTTAATA<br>TTTTTATGTTAGCAACCATGAGAGACGCAGAGAGAGAGACAGAAAGATGAAAAAGGGCGC<br>TTGCGGACGTCGTCGGGTAGGGTGCCTGAAGTCGATGCGAAGAGATGGGAGTGGAGAGAG<br>TGTGGTCACCATGAGAGACGCAGACATGTACACACATACTACCCTTT
**CS1**	**IIIL**	GGCGAATTTTGCGCTCAGAACGCAGACTCAGTTCAGACTCGAAAGTTATTAATCCTTGAAA<br>TTTCATAGGATTTCGCGCTTTTAGGCTTAGGCTTAGGCTTAGGCTTAGGCTTAGGCTTAGGC<br>TTAGGCTTAGGCTTAGGCTTAGGCTTAGGCTTAGGCTTAGGCTTAGGCTTAGGCTTAGGCTT<br>AGGCTTAGGCTTAGGCTTAGGCTTAGGCTTAGGCTTATGAAAAATAGAAAAATCTTACTTA<br>TCCCCCACGTATTCTCCACTCTGCTCAAGAAAAGTTCTTTAGCCAGCTCCTTTAATTGTTCC<br>AGCGCCTTCGAATGATCCAAAGCAACCAGAATAACTTTTTTCCCAGACTTGTGAATAAACG<br>TCACAGAAACCAGATCGGTTCCGTCGTCTCTACGACCACATGCTC
**NS1**	**VR**	TAAGCCTAAGACTAAGCCTAATACTAAGCCTAAGCCTAAGACTAAGCCTAAGCCTAATACT<br>AAGCCTAAGCCTAAGACTAAGCCTAATACTAAGCCTAATACTAAGCCTAAGCCTAAGACTA<br>AGCCTAAGCCTAAGCCTAAGCCTAAGCCTAAGCCTAAGCCTAAGCCTAAGCCTAAGCCTAA<br>GCAAATCCCCAGAAAGGTGGTTCAGTACCTTACGCAAGTAGCTGGTTTTTGTTCAGTAATT<br>TAGAAATCAAGAAACTTCGCGGAATTAAAGAATTTTCAAAATAGCTTGAAAACATTCAATA<br>AACCTTTATGCTATTAAGGATGCCAATAAGTTACAGATAA
**NS1**	**XR**	CTCGACTCCGTGCACGAGGGCACCGAGGAAAGATAGCCACACGTAGCCGAGGTAGACAAT<br>GAACATGCAGGCGGCAATGAACACAAGGCGGAATGGTGCGGCAAGTTCTCCGAGAATCGG<br>GTACGCGCAGAAGCCAGAAGTGTAGAAGACGTAATGGAGGCTGGAAAATGGTGTGAGGA<br>AGGCAAAATTAGATGTAGGCGCCAAATATCTAGAGGCTTCACTAAAAAAAAAACGTTTTG<br>ATTTGGCCATGAAAACGATTTTGAAAAAGGAAAATGTTTTGCTATCGCAATTTTTTAGGCTT<br>AGGCTTAGGCTTAGGCTTAGGCTTAGGCTTAGGCTTAGGCTTAAGCTTAAGCTTAAGCTTA<br>GGCTTAGGCTTAAGGCTTAGGCTTAGGCTTAGGCTTAGCTTAAGCTTAGGCTTAAGTCTTA<br>GGCTTATCTTAGGCTTAGGCTTAGACTTAGGC
**NS1**	**IIL**	CGACCCGTCATCGTTTACAGGAGCATATAGGTTGGCAGGCAGGCAAAATTAGAGGTACCC<br>GCCAAATATCTAGAAGCTTCACTAAAAAAAAAACGTTTTGAATTTAGCATGAAAACAATTT<br>TGAAAAAGTAAAATGTTTTCTTATCTCAATTTTTTAGGCTTAGGCTTAGGCTTAGGCTTAGG<br>CTTAGGCTTAGGCTTAGGCTTAGGCTTAGGCTTAGGCTTAGGCTTAGGCTTAGGCTTAGGCT<br>TAGGCTTAGGCTTAGGCTTAGGCTTAGGCTTAGGCTTAGGCTTAGGCATAGGCATAGGCAT<br>AGGCATAGGCATAGACATAGGCATAGGCATAGGCATAGGCATAGGCATAGGCATAGGCAT<br>AGGCATAGGCATAGGCATAGGCATAGGCATAGGCATAGGCATAGGCATAGGCATAGGCAT<br>AGGCATAGGCATAGGCATAGGCATAGGCATAGGCATAGGCATAGGCATAGGCATAGACAT<br>AGGCATAGGCATAGGCATAGGAATAGACATAGGCATAGGCATAGGCATAGGCATAGACAT<br>AGGCATAGGCATAGGCTTAGGCTTAGGCTTAGTATTAGGCTTAGGCTTAGGCTTAGGCTTA<br>GGCTTAGGCTTAGTCTTAGGCTTAGGCTTAGTATTAGGCTTAGGCTTAGTATTAGGCCTAAA<br>CACAGGAGCATATAGGTTGGCAGG

* Blue, proximal telomere region sequences; Red, TALT sequence; Yellow, Telomere repeat

Supplementary Table 3. The list of putative TALT regions

Putative T-ALT regions				Upstream telomere sequence			Downstream telomere sequence			Gene information	
chr	start	end	size (bp)	size (bp)	log odds score	strand	size (bp)	log odds score	strand	gene	type
chrI	432	613	181	telomere	58.1	-	270	37.4	-	none	intergenic
chrI	833772	834061	289	336	44.3	+	336	44.3	+	ZC123.3	intronic
chrI	834301	834428	127	336	44.3	+	56	51.2	+	ZC123.3	intronic
chrI	939858	940049	191	99	51.2	+	81	37.4	+	Y95B8A.2.1	intronic
chrI	940129	940702	573	81	37.4	+	177	44.3	+	Y95B8A.2.1	exonic
chrI	940878	942562	1684	177	44.3	+	112	51.2	-	Y95B8A.2.1; Y95B8A.1	exonic
chrI	942673	943271	598	112	51.2	-	136	37.4	-	Y95B8A.1	intronic
chrI	2368870	2370727	1857	177	37.4	+	161	58.1	-	Y39G10AR.3	exonic
chrI	11450507	11451192	685	112	58.1	-	131	44.3	+	ZK1025.2	exonic
chrI	11470222	11470907	685	131	44.3	-	118	58.1	+	ZK1025.8	exonic
chrI	11933566	11934647	1081	224	58.1	+	145	37.4	+	none	intergenic
chrI	11948721	11949762	1041	187	37.4	+	124	37.4	+	R06C1.6	intronic
chrI	13903320	13903742	422	115	37.4	-	194	51.2	-	Y71A12B.4	intronic
chrI	13903935	13904257	322	194	51.2	-	195	44.3	+	Y71A12B.4	intronic
chrII	1241835	1242099	264	46	37.4	-	79	37.4	-	F47F6.1b	intronic
chrII	2633601	2634817	1216	87	37.4	-	58	44.3	+	F22E5.10	exonic
chrII	2846633	2846821	188	66	44.3	-	66	37.4	-	Y110A2AL.9	intronic
chrII	2846886	2847760	874	66	37.4	-	285	37.4	-	Y110A2AL.9	exonic
chrII	2978218	2979033	815	186	37.4	+	410	44.3	-	K08A2.5a.1	exonic
chrII	3014268	3015385	1117	244	51.2	-	95	44.3	-	C41H7.2	exonic
chrII	4105555	4106577	1022	44	37.4	+	87	44.3	+	none	intergenic
chrII	4152342	4153478	1136	98	37.4	+	194	44.3	-	none	intergenic
chrII	4153478	4155197	1719	194	44.3	-	286	44.3	+	none	intergenic
chrII	11928513	11930216	1703	256	44.3	+	147	44.3	-	Y17G7A.3; W03C9.4a	exonic
chrII	11930362	11931388	1026	147	44.3	-	130	37.4	+	W03C9.4a	intronic
chrII	11931517	11932237	720	130	37.4	+	212	44.3	+	W03C9.4a	exonic
chrII	12169744	12169861	117	255	37.4	+	140	37.4	+	Y57A10A.8	intronic
chrII	13974368	13975464	1096	89	44.3	+	61	44.3	+	Y39G8B.2	exonic
chrII	14117024	14117146	122	94	37.4	-	64	37.4	-	K09E4.4.1	intronic
chrII	14206525	14208275	1750	275	37.4	-	309	37.4	+	Y48B6A.8	exonic
chrII	14819105	14819612	507	44	44.3	-	262	44.3	-	W01D2.2b.1	intronic
chrII	15253799	15254879	1080	107	51.2	-	673	37.4	+	Y46E12BL.5	exonic
chrIII	1256332	1257439	1107	118	44.3	+	239	37.4	+	Y116D3B.5;Y119D3B.19	exonic
chrIII	1264771	1265880	1109	116	44.3	+	239	37.4	+	Y119D3B.3;ZC47.7	exonic
chrIII	1717644	1718805	1161	246	51.2	-	309	37.4	+	Y22D7AR.12	exonic
chrIII	11784827	11784950	123	95	58.1	-	59	37.4	-	C24H11.3	intronic
chrIII	12130560	12131640	1080	159	44.3	-	496	37.4	+	none	intergenic
chrIII	12640809	12642321	1512	203	37.4	+	605	37.4	+	Y11B2A.14a.2	exonic
chrIII	12944443	12945013	570	122	44.3	-	216	44.3	+	Y37D8A.23a	exonic
chrIV	1069499	1071380	1881	66	37.4	-	97	58.1	+	none	intergenic
chrV	1230093	1230609	516	137	37.4	+	272	37.4	-	C39F7.2a	intronic
chrV	1318725	1318828	103	179	44.3	-	87	44.3	-	none	intergenic

chrV	1480585	1481676	1091	187	37.4	-	255	37.4	-	none	intergenic
chrV	1481930	1483211	1281	255	37.4	-	354	51.2	+	C38C3.4a	exonic
chrV	2708090	2709410	1320	667	37.4	-	305	37.4	-	C24B9.3a	exonic
chrV	16593211	16594103	892	220	37.4	+	104	37.4	+	T03E6.7.1	exonic
chrV	18566044	18566380	336	420	58.1	-	267	51.2	-	none	intergenic
chrV	18638371	18639669	1298	325	44.3	-	175	58.1	+	Y69H2.14.2	exonic
chrV	19229625	19231091	1466	584	44.3	+	451	37.4	+	T25H2.5	exonic
chrV	19231541	19232568	1027	451	37.4	+	229	51.2	-	T25H2.5; T26H2.12	exonic
chrV	19722468	19723307	839	75	37.4	-	32	37.4	+	Y116F11A.9	exonic
chrV	19827792	19829288	1496	223	44.3	-	352	44.3	+	Y116F11B.3.1	exonic
chrV	20001755	20001897	142	75	37.4	+	105	44.3	+	Y60A3A.1.1	intronic
chrV	20002001	20002293	292	105	44.3	+	558	37.4	+	Y60A3A.1.1	exonic
chrX	1836504	1836853	349	208	37.4	-	531	37.4	-	T26C11.2	exonic

The two shaded regions are TALT elements identified in this study

Supplementary Table 4. The list of RNAi-subjected genes, predicted to have a role in ALT

Gene name	Predicted roles	Gene name	Predicted roles
atl-1	DSB response	*lin-35*	RB homolog, tumor suppressor
atm-1	DSB response	*mlh-1*	mismatched repair
brc-1	Recombination	*mre-11*	DSB response
brc-2	fanconi anemia pathway	*msh-2*	mismatched repair
ceh-37	Telomere binding protein	*msh-6*	mismatched repair
cep-1	P53 homolog	*mus-81*	endonuclease
chk-2	Meiotic recombination	*plp-1*	Telomere binding protein
com-1	Meiotic recombination	*pms-2*	mismatched repair
D1081.9	Meiotic recombination	*pot-1*	Telomere binding protein
dna-2	helicase	*prom-1*	Meiotic recombination
dog-1	helicase	*rad-50*	Homologous recombination
drh-3	fanconi anemia pathway	*rad-51*	Homologus recombination
exo-3	AP endonuclease	*rad-54*	Homologous recombination
F55A12.10	Meiotic recombination	*rfs-1*	Homologous recombination
fanci-1	fanconi anemia pathway	*rpa-1*	RPA, replication
fcd-2	fanconi anemia pathway	*rpn-1*	protease
hel-308	helicase	*rtel-1*	helicase
him-17	DSB formation	*set-11*	Histone methyltransferase
him-18	Homologous recombination	*set-25*	Histone methyltransferase
him-3	Meiotic recombination	*spo-11*	DSB formation
him-6	BLM helicase	*top-3*	Topoisomerase IIIa
him-8	Meiotic recombination	*vhp-1*	MAPK
hmg-5	Telomere binding protein	*wrn-1*	WRN helicase
hpl-2	Heterochromatin protein 1	*xpf-1*	ERCC1/XPF endonuclease
hrp-1	Telomere binding protein	*Y39B6A.16*	Meiotic recombination
ku-80	Non-homologous end joining	*zhp-3*	Meiotic recombination

Supplementary Table 5. Primers used in this study

Primer name	Sequence (5' to 3')	Figure
trt-1 deletion F	CCGCCATATGTCCACTTTTT	Supplementary Figure 1c
trt-1 deletion R	TCCCTGTGGAACTCCTTTTG	Supplementary Figure 1c
TALT1 F*	GAAGGATGGGATGGAACTGA	Figure 2c, 4b<br>Supplementary Figure 4c, 7c
TALT1 R	AACAAGCTTCTGGCACGTCT	Figure 2c, 4b<br>Supplementary Figure 4c, 7c
TALT2 F*	GTTTTCATGCTAAATTCAAAACG	Figure 4e<br>Supplementary Figure 4f
TALT2 R	AAACACAGGAGCATATAGGTTGG	Figure 4e<br>Supplementary Figure 4f
TALT2 O	GCGAAGGTAGTGCACATCAA	Figure 4e
Chromosome I R	GTGCGGGGAGGGAGTATTA	Figure 2c
Chromosome II L	CGACCCGTCATCGTTTACA	Supplementary Table 2
Chromosome II R	CGATGCGAAGAGATGGGAGT	Figure 2c
Chromosome III L	GCCTAAAAGCGCGAAATCCT	Figure 2c
Chromosome V R	CCCACCGGAAACGAATAAAT	Supplementary Table 2
Chromosome X R	TTTCCAGCCTCCATTACGTC	Supplementary Table 2

* TALT1 F and TALT1 R for TALT1 probe and TALT1 qPCR
* TALT2 F and TALT2 R for TALT2 probe and TALT2 qPCR
